# Short-Term and Long-Term Effects of Inhaled Ultrafine Particles on Blood Markers of Cardiovascular Diseases: A Systematic Review and Meta-Analysis

**DOI:** 10.3390/jcm14082846

**Published:** 2025-04-21

**Authors:** Joanna Izabela Lachowicz, Paweł Gać

**Affiliations:** Department of Environmental Health, Occupational Medicine and Epidemiology, Wroclaw Medical University, Mikulicza-Radeckiego 7, 50-368 Wroclaw, Poland; pawel.gac@umw.edu.pl

**Keywords:** ultrafine particles (UFPs), blood biomarkers, cardiovascular diseases (CVDs), inflammation

## Abstract

**Background/Objectives:** Air pollution is the highest environmental risk factor of mortality and morbidity worldwide, leading to over 4 million deaths each year. Among different air pollutants, ultrafine particles (UFPs) constitute the highest risk factor of cardiovascular diseases (CVDs). Epidemiological studies have associated UFPs with the short- and long-term imbalance of numerous blood markers. Our objective was to systematically review the short-term and long-term impact of UFP exposure on blood markers of CVDs. **Methods:** We prepared the systematic review of CVD blood markers and meta-analyses of the short- and long-term effects of UFP exposure on high-sensitivity C-reactive protein (hsCRP) concentration. The eligibility criteria were established with the use of the Provider, Enrollment, Chain, and Ownership System (PECOS) model, and the literature search was conducted in Web of Science, PubMed, and Scopus databases from 1 January 2013 to 9 January 2025. The risk of bias (RoB) was prepared according to a World Health Organization (WHO) template. **Results:** The results showed an increase in hsCRP as a result of both short-term and long-term UFPs. Moreover, IL-6 and IL-1β together with other inflammatory markers increased after short-term UFP exposure. In addition, different nucleic acids, among which were miR-24-3p and let-7d-5p, were differentially expressed (DE) as a result of short-term UFP exposure. Chronic exposure to UFPs could lead to a persistent increase in hsCRP and other blood markers of CVDs. **Conclusions:** Our findings underline that UFPs may lead to the development and/or worsening of cardiovascular outcomes in fragile populations living in air-polluted areas.

## 1. Introduction

Air pollution constitutes the highest environmental risk factor of mortality and morbidity worldwide [1]. According to the Global Burden of Disease (GBD) report, air pollution led to 120 million disability-adjusted life years (DALYs) and 4.72 million deaths in 2021 [2]. Ambient air pollution has been correlated with a number of diseases, among which are respiratory diseases, decreased lung function, cancer, obesity, diabetes, and cardiovascular diseases (CVDs) [3,4,5]. According to the GBD report published in 2015, air pollutants contributed to 19% of all CVD deaths [1].

Among different air pollutants (nitric oxides (NO_x_), sulfate oxides (SO_x_), ozone (O_3_), carbon oxides (CO_x_), black (BC) or elemental carbon (EC), volatile organic compounds (VOCs)), particulate matter (PM_x_) of different diameter sizes constitutes the high risk of the development of different diseases [3]. Moreover, the size of PMs has been inversely correlated with their potential risk to human health [3].

Ultrafine particle (UFP) or PM_0.1_ (PM < 0.1 µm diameter) exposure is considered the highest risk factor for cardiovascular diseases (CVDs) due to the high particle number concentration (PNC), reactive surface (e.g., pro-oxidative), and high surface/mass ratio, which together with high solubility and charge increase the alveolar penetration and systemic circulation, leading to damage of various tissues [6,7].

Biomarkers are defined as “a characteristic that is objectively measured and evaluated as an indication of normal biologic processes, pathogenic processes, or pharmacologic responses to a therapeutic intervention” [8,9]. They must be (1) accurate, (2) reliable, and (3) therapeutic (impact with early intervention) [9]. Nowadays, biomarkers are fundamental in the diagnosis, prognosis, and decision making in the management of cardiovascular pathologies.

Blood pressure (BP) and heart rate variability (HRV) measures are two well-established practical, noninvasive tools to quantify cardiac dysregulation in hypertension [10,11]. Blood biomarker analysis can complete the whole picture of CVD stage, progression, and prognosis. Inhaled UFPs enter deep into the respiratory tract, leading to significant health impacts, from weak temporary respiratory difficulties to lung cancer, central nervous system pathologies, DNA mutations, and even cardiovascular and respiratory mortality. UFPs are characterized by a large surface area, over which particles can transport a high number of toxic agents that cause tissue and cell injury, generating oxidative stress and inflammation states.

The well-known blood biomarkers of CVDs are related to specific pathophysiological processes, among which are myocardial necrosis, inflammation, plaque instability, platelet activation, neurohormonal activation, and myocardial stress [12].

Previous systematic reviews on UFPs and CVDs focused on physical exams of cardiac imbalance, namely BP and HRV, as risk predictors in populations exposed to UFPs. Despite there being numerous studies on CVD blood marker imbalance due to UFP exposure, there is still significant inconsistency in the choice of experimental protocols determining and quantifying UFP exposure as a risk factor of CVDs.

We conducted this systematic review and meta-analysis to provide quantitative estimates of short-term and long-term ambient UFP effects on CVD blood marker indices. We aimed to list the most CVD-specific biomarkers, which together with BP and HRV exams could deliver more precise and accurate analysis of UFP exposure as a risk factor of CVDs. Among the selected records, high-sensitivity C-reactive protein (hsCRP) was the most representative blood biomarker of inflammation, and was analyzed in the highest number of studies. There is growing evidence that differential analysis of nucleic acids could be a valuable tool in the analysis of UFP exposure effects on health.

## 2. Methods

The systematic review with meta-analysis presented here was written following the PRISMA 2020 statement [13].

### 2.1. Eligibility Criteria

The selection of the records was decided with the use of the PECOS model (Tables S1 and S2). The records were recovered with the use of the following keywords: “nanoparticles” (and its synonym: “ultrafine particles”), “cardiovascular” (the principal outcome of interest), “exposure” (as the main reason of the outcome), and “blood markers”. Exclusively experimental research studies were chosen for this systematic review. Reviews (and systematic reviews), book chapters, meta-analyses, comments, and letters were not taken into account. Neither studies from conference proceedings nor gray literature were included in this study. Only studies published in peer-review journals with a clear and transparent revision process were selected in order to avoid low-quality data with a high risk of bias (RoB). In this study, we focused only on studies with human participation (e.g., cohort studies, crossover studies). Studies with animal participation, as well as in vitro and ex vivo cell models, were not taken into account in this systematic review. Numerous animal and cellular models have provided mechanistic insights into UFP hazards and effects on blood marker increase, and have been reviewed extensively elsewhere (i.e., Li et al. [14]).

Records satisfying the following criteria were taken for this study: (1) epidemiological studies investigating at least one blood marker; (2) studies measuring UFPs with a dimension size of ≤10 nm; (3) records on short-term effects (lag ≤ 7 days); (4) records on long-term effects (lag ≥ 7 days); (5) records presenting quantitative analysis of association measures with 95% confidence intervals (CIs) or standard errors from single-pollutant models; and (6) records reported in the English language. Records in different languages were not included in order to avoid insufficient data comprehension and possible data misinterpretation due to an incorrect automatic translation process.

The selected studies analyzed the relationship between UFPs and blood marker imbalance. In the systematic review presented here, both records with the data presenting and not presenting statistically significant associations among UFPs and selected blood markers were considered. However, only records quantifying the particle number concentration (PNC) were analyzed. This study focused on the short- and long-term effects of UFP exposure leading to blood marker (nucleic acids, proteins and peptides, lipids) imbalance. Thus, only records with high-quality results on blood marker imbalance as a result of UFP exposition were examined in this review.

### 2.2. Information Sources

The data were searched in four databases: Web of Science, Embase, PubMed, and Scopus. The data research was based on keywords, titles, abstracts, and, when necessary, full texts. The last data search was made on 9 January 2025.

### 2.3. Search Strategy

The records were retrieved with the use of the following keywords: “nanoparticles” OR “ultrafine particles” AND “cardiovascular” AND “exposure” AND “blood marker”. The selected data were published in English in a time range from 1 January 2013 to 9 January 2025.

### 2.4. Selection Process

Joanna Izabela Lachowicz and Pawel Gac independently selected the articles and assessed each record’s eligibility. Studies that had the following standards were chosen for this systematic review: (1) epidemiological data analyzing blood markers; (2) records monitoring the PNC for UFPs with a diameter lower than 100 nm; (3) studies analyzing short- and/or long-term UFP effects on blood marker imbalance; and (4) records calculating the grade of associations with 95% CIs or standard errors calculated with single- and multiple-pollutant models.

Records which did not quantify effect estimates standardizable for meta-analysis were not included in this study.

### 2.5. Data Items and Data Collection Process

The data extraction process was conducted following the instructions of Pedder et al. [15] and Jonnalagadda et al. [16]. In brief, Joanna Izabela Lachowicz and Pawel Gac independently selected the data from the studies with the use of a Microsoft Excel template: (1) name of the first author and date of publication; (2) study protocol; (3) outcome and exposure data, together with proper statistics data; and (4) effect estimates of the PNC.

### 2.6. Study Risk of Bias Assessment

In this study, the RoB was assessed with the use of a tool developed by the WHO [17]. In brief, the RoB was evaluated in different areas (Table S3): confounding, selection bias, exposure assessment, outcome measurement, and missing data. Every area is divided into four domains. Joanna Izabela Lachowicz and Pawel Gac assigned independently the RoB for each domain as “low” risk, “some concerns”, or “high” risk and justified all scores in a Microsoft Excel template. The “high” score was given when the significant data were missing or if the data were not assigned correctly. The “some concerns” score was assigned if data were not present in the main text or its references.

The overall score was assigned “high” if any domain had a “high” RoB score. The overall rate was rated as “some concerns” if any domain had “some concerns” and no domain was assigned as “high”. An overall score was “low” if all domains were assigned as “low”. Discordances were resolved through discussion between Joanna Izabela Lachowicz and Pawel Gac. Moreover, additional consultations with the research group of Wroclaw Medical University were conducted to avoid any discordances and improper data evaluation.

### 2.7. Sensitivity Analysis

We made the analyses to evaluate the eventual causes of heterogeneity and examine the robustness of the outcome evaluations: (1) We limited our meta-analyses to hsCRP concentration data with 95% CIs. (2) We analyzed records, which assessed the PNC of UFPs sized ≤100 nm. (3) We independently analyzed short- and long-term effect estimates on hsCRP. (4) In all data elaborations, we chose the outcome estimate that had the highest significance for each study group and showed an imbalance in hsCRP concentration in correlation with a high PNC.

Statistica (StatSoft Polska Sp. z o.o. 2024; Zestaw Plus Version 5.0.96; www.statsoft.pl last access on 1 April 2025), Microsoft Excel Office 360 software, and Grapher 7.01870 Golden software were applied for statistical calculations and proper diagram representation of the analysis.

In the study with meta-analysis presented here, we centered attention on the short- and long-term effects on CVD blood markers. Thus, the sensitivity analysis took out other features, which could influence the heterogeneity of the overall data. It is worth noting that the experimental protocols among different records vary significantly in terms of the studied populations, numerosity of the enrolled population, and type of technique used for PNC determination. Moreover, the PNC and UFP origin vary among studies.

The PNC precision and accuracy are strongly influenced by the quantitative method chosen by the authors. Computational modeling of air pollutants (among which are UFPs) chosen for the cohort studies covers long periods and wide areas. However, it is less accurate and precise than air monitoring systems or personal PNC counters chosen for cross-sectional studies. Personal PNC counters are a much more precise and accurate method to analyze indoor and outdoor PNCs than computational models. Indeed, personal counters were selected for short-term studies with participation of low-number populations. The selected studies were conducted in different countries on different continents and in areas of varying industrialization. Thus, the baseline PNC was significantly different among the analyzed studies (Table 1).

### 2.8. Effect Measures

The effect outcomes presented as the mean, % changes, or β-estimates in blood markers (95% CIs) were taken from the original manuscript. The data selected for meta-analysis of hsCRP were expressed as percentage changes (95% confidence interval) and were found in the original manuscript. Whenever the final outcomes were expressed in different formats, the correct standardization was made following the previously published procedures (Supplementary Materials, Text S2) [11]. We made meta-analyses on hsCRP data when a minimum of three effect estimates were found for a short- or long-term period. We chose the lag time presenting the outcome with the highest significance per study participants, in spite of the effect trend.

The Q-test and I^2^ were executed to analyze heterogeneity between selected records following the Neyeloff et al. method [42]. The funnel plots with Egger’s and/or Begg’s test analysis were not made due to a low number of records (a minimum of 10 records are needed for each test). The mean effect estimates of hsCRP concentrations were taken from the articles. Absent data were retrieved from the graphics present in the main text or Supplementary Materials with the use of the PlotDigitizer platform (PlotDigitizer: https://plotdigitizer.com/app; last access on 21 January 2025).

### 2.9. Synthesis Methods

The records examining blood marker imbalance in adult volunteers were used for the overall blood marker analysis and hsCRP meta-analysis. There was one record [43] studying systemic inflammation after UFP exposition in infants, and it was not used in the final analysis nor meta-analysis, while hsCRP baseline mean values in children were significantly different than values in adult participants of other studies. One record [19] was excluded from the meta-analysis of hsCRP because the provided data were not sufficient to calculate the percentage changes (95% confidence interval).

## 3. Results

### 3.1. Study Selection

**Figure 1:**
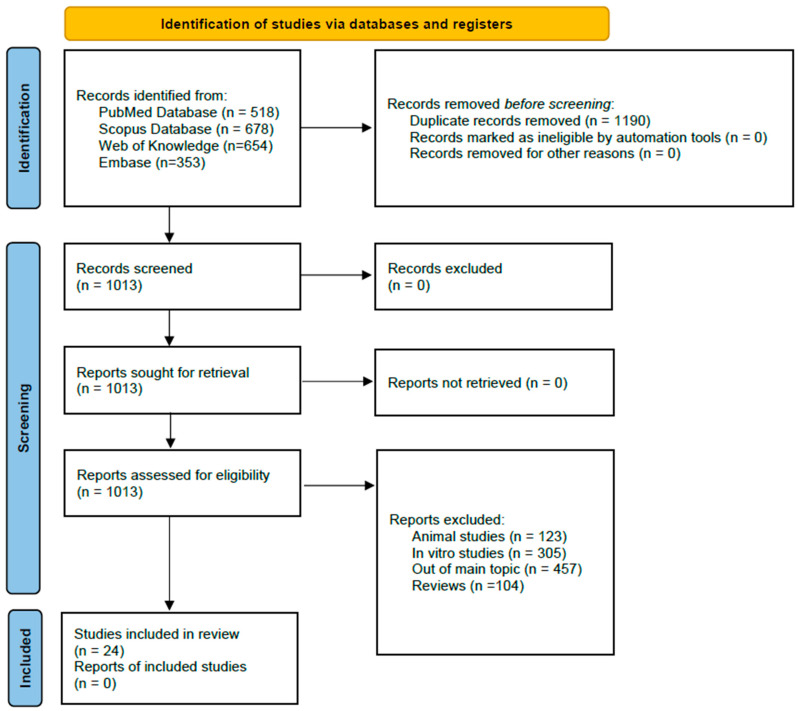
Flowchart of the record screening process following PRISMA 2020 [13].

The literature search in the time range January 2013–December 2024 in four databases (Embase, PubMed, Scopus, and Web of Science) presented here delivered 2203 records (Figure 1). After removing 1190 duplicates, we analyzed 1013 records and selected 24 eligible articles, which were further used for CVD blood marker analysis. Table 1 summarizes the main characteristics of the 24 records used for the blood marker systematic review analysis.

### 3.2. Study Characteristics

The 24 selected studies were conducted in North America (6), the European Union (10), and Asia (8). Among the twenty-four studies, only one study was executed in laboratory conditions with controlled UFP exposure. The remaining studies were driven in real-word conditions, among which there were four occupational studies [19,25,33,34].

Most of the analyzed studies were conducted with crossover protocols (nine studies). There were seven cross-sectional studies and four panel studies. Moreover, three longitudinal and one cohort studies were selected for the analysis.

#### 3.2.1. Characteristics of Short-Term Effect Studies

There were 18 studies selected for the analysis of the short-term effects of UFP exposure on blood markers of CVDs. Among the selected studies, five studies used panel protocols [19,26,32,33,41], four studies were conducted with the cross-sectional method [20,24,25,34], eight studies were made in the crossover regime [23,28,30,31,36,39], and there was one cohort study [38].

The panel study conducted by Meier at al. [19] focused on the association of air pollutants (UFPs, PM_2.5_, CO, NO_2_, and O_3_) and noise with biomarkers of respiratory and cardiovascular conditions in a cohort of 18 healthy male highway maintenance workers. The PNC was measured with the use of a personal monitoring system for both indoor and outdoor measurements. BP, proinflammatory (hsCRP, IL-7, TNF-α, SAA) and prothrombotic (plasma vWF and tissue factor) blood markers, as well as lung function and fractional exhaled nitric oxide (FeNO) were measured approximately 15 h after work. Moreover, HRV was monitored during sleep hours (10 h after work). The authors concluded that exposure to particulate matter air pollutants and noise during highway maintenance might increase cardiovascular health risk. Exposure to PM_2.5_ was positively associated with hsCRP and SAA and was negatively associated with TNF-α.

Liu et al. [26] were interested in the association of outdoor and indoor UFP exposures with cardiopulmonary health effects in 100 adult subjects in the urban areas of northern Taiwan. Blood pressure and spirometry (FEV1) were used to evaluate the cardiovascular burden, while blood hsCRP concentration was an indicator of inflammation. According to the presented data, UFP exposure significantly increased blood pressure and hsCRP levels, PM_2.5_ and NO_2_ were associated only with blood pressure increase, and O_3_ was correlated with decreased lung function.

Mancini et al. [32] examined the correlation between PM_2.5_ and UFP and expression levels of peripheral blood miRNAs in the healthy subjects living in four European cities (Basel, Norwich, Turin, and Utrecht). The authors presented a list of the miRNA sequences in which the expression changes with growing PM exposure.

Also, Guo et al. [33] were interested in the differential expressions of nucleic acids due to UFP exposure in toner-based printing equipment manufacture. Moreover, protein expression profiles were analyzed. The obtained results showed that workers exposed to particulate matter have DE RNA related to inflammatory and immune responses, metabolism, cardiovascular impairment, neurological diseases, oxidative stress, physical morphogenesis/deformation, and even tumors.

In the last selected panel study, Jiang et al. [41] investigated the cardiovascular responses to short-term UFP exposure and the biological pathways studied with the participation of 32 healthy volunteers living in Shanghai (China). According to the authors, UFP exposure led to systolic BP (SBP) and a pulse wave velocity increase within 24 h. Moreover, significant changes were observed in proteomic and metabolomic data related to systemic inflammation, oxidative stress, endothelial dysfunction, coagulation, and in lipid transport and metabolism.

The cross-sectional model of epidemiological observations was applied by four research groups. Karottki et al. [20] studied lung and cardiovascular imbalance in correlation with outdoor and indoor exposure to both fine and UFP matter in middle-aged populations living in Copenhagen (Denmark). Moreover, bacteria, endotoxins, and fungi were analyzed in material from electrostatic dust-fall collectors in the selected residences. Indoor UFP exposure was associated with HbA1c (the prediabetic marker) and systemic inflammatory markers (leukocytes and monocytes), while hsCRP was correlated with indoor PM_2.5_. Dysregulated expressions of adhesion markers on monocytes and lower lung function were related to indoor endotoxin presence.

Fuller et al. [24] recruited participants from three areas based on residential distance to the highway. The aim of the study was to investigate the correlation between UFP exposure and the response of biomarkers of inflammation and coagulation. In general, PNC increase was positively correlated with longer averaging times for IL-6, hsCRP, and fibrinogen. Of note is that the authors did not observe trends between the PNC and biomarkers in relation to domiciles.

Shvedova et al. [25] conducted analysis of ncRNA and mRNA dysregulated expression in 15 healthy workers exposed to carbon nanotubes. There was a set of miRNAs and their target genes with roles in cell cycle regulation/progression/control, apoptosis, and proliferation dysregulated profiles in the group of exposed workers. The identified pathways and signaling networks also revealed carbon nanotubes’ potential to initiate pulmonary, carcinogenic, and cardiovascular outcomes in humans.

Bello et al. [34] examined systemic inflammation from copier-emitted particles in 19 healthy operators at six Singaporean workplaces. Among the 14 inflammatory cytokines in plasma and nasal lavage, IL-1β, IL-1 (nasal lavage), fractalkine, IL-1β, TNF-α, and IFN-γ in plasma were strongly and positively associated with at least one exposure metric (PM_2.5_, UFP, and/or LDSA), whereas the granulocyte colony-stimulating factor was negatively associated.

The crossover method was the most popular among the selected studies on UFPs’ short-term effect on inflammatory blood markers of cardiovascular diseases. Devlin et al. [21] chose for their study the population with metabolic syndrome, exposing them for 2 h to clean air and then for 2 h on concentrated ambient UFPs. They could observe in the whole population the decrease in blood plasminogen and thrombomodulin, while hsCRP and SAA increased after exposure to UFPs.

Karottki et al. [23] enrolled elderly subjects for a four-week home air filtration study in Copenhagen. Each participant was randomly assigned to consecutive two-week periods with or without the inclusion of a high-efficiency particle air filter in re-circulating custom-built units in their living room and bedroom. The data showed inverse associations between microvascular function and outdoor PNC; granulocyte counts and PM_2.5_; CD31 expression and dust fungi; and surfactant protein-D and dust endotoxin.

Kumarathasan et al. [28] studied emission-related effects on healthy volunteers who spent five days near a steel plant and at a well-removed college site. The authors observed that air pollution exposures were associated with increased pro-inflammatory cytokines (e.g., IL-4, IL-6) and endothelins. Moreover, plasma IL-1β and IL-2 were increased after exposure. Interquartile range (IQR) increases in CO, UFP, and SO_2_ were correlated with growing plasma pro-inflammatory cytokines (e.g., IL-6, IL-8) and ET-1(1–21) concentrations. Additionally, hsCRP was positively associated with increased heart rate.

In the study by Espin-Pérez et al. [30], volunteers were asked to walk on the busy streets of London or Barcelona for two rush hours, and on a different day for the same time in the park areas. Transcriptomics and miRNAs were analyzed in blood samples. Five different miRNAs (hsa-miR-197-3p, hsa-miR-29a-3p, hsa-miR-15a-5p, hsa-miR-16-5p, and hsa-miR-92a-3p) previously linked to air pollution-related tumors and Alzheimer’s disease were associated with even low exposure periods and air pollutant levels.

Krauskopf et al. [31] analyzed miRNA plasma samples from healthy volunteers or ischemic heart disease or chronic obstructive pulmonary disease patients. The participants were asked to walk for 2 h along a high-traffic street in London, and in a separate session they were asked to walk in a park area. The authors identified 54 circulating miRNAs that were dose-dependently correlated with air pollutant concentrations.

Du et al. [35,39] studied plasma-derived exosomal miRNA and lncRNA changes following 4 h exposure to traffic-related air pollution in road and park sessions in London. There were 271 exosomal miRNAs (212 upregulated and 59 downregulated; in particular, miR-3612, miR-21-5p, and miR-195-5p) as well as 55 lncRNAs (including lncRNA NORAD, MALAT1, and H19) that were significantly associated with air pollutants.

Zhang et al. [36] conducted the crossover trial in 56 young, healthy adults, who were engaged in two 4 h exposure sessions on a main road or in a park. After TRAP exposure they observed increased BP, while HRV was decreased. Moreover, there were different molecular alterations in systemic inflammation, oxidative stress, endothelial dysfunction, coagulation, and lipid metabolism in all participants.

The only cohort study used to investigate the short-term effects of UFP exposure on inflammatory blood markers was conducted by Roswall et al. [38]. To investigate associations between short-term exposure to air pollution and high-density lipoprotein (HDL), non-high-density lipoprotein (non-HDL), and BP, over thirty thousand participants of the Danish Diet, Cancer and Health—Next Generations cohort were enrolled. Air pollutants were adversely associated with lipid profile and BP. Moreover, the strongest associations were found among overweight and/or obese participants.

#### 3.2.2. Characteristics of Long-Term Effect Studies

Among the records selected for the analysis of the long-term effects of UFP exposure to inflammation blood markers there were three cross-sectional studies, two longitudinal studies, and one crossover study.

Brugge et al. [18] conducted a cross-sectional study of inflammatory blood markers in populations living close to a highway and around Boston (MA, USA). They could observe that hsCRP and IL-6 differed by distance category relative to urban background. However, there was a weak association for TNF-RII and fibrinogen.

Pilz et al. [29] cross-sectionally analyzed data of 2252 participants of the German KORA baseline survey without health status discrimination. Single-pollutant models for different air pollutants presented a positive (but non-significant) correlation with hsCRP. In the case of PNC, there was a slight increase in hsCRP when the PNC concentration was getting higher. Of note is that the estimated effects were higher for women, participants without diabetes and without a history of CVD, and non-obese and non-smoking volunteers.

Vogli et al. [40] examined the association between long-term exposure to air pollutants and blood biomarkers of inflammation and coagulation in the German KORA S4 cohort study (3969 participants, and a subsample of 1433 older participants) without health status discrimination. The results showed that fibrinogen and hsCRP increases were associated with UFP exposure, while adiponectin decrease was correlated with an increase in PM_2.5_. Moreover, in the older participant subsample, PM_2.5_ was associated with higher IL-6 concentrations.

Padró-Martínez et al. [22] carried out a study analyzing high-efficiency particulate arrestance (HEPA) filtration in houses placed in the proximity of a highway. Volunteers were randomly exposed to filtered air for 21 days and unfiltered air for the same period. The results showed insignificant differences in blood pressure or the biomarkers hsCRP, fibrinogen, and TNF-RII measured after exposure to HEPA-filtered air compared to measurements after exposure to sham-filtered air.

Corlin et al. [27] examined the data of 791 adults (without health status discrimination) from the longitudinal Boston Puerto Rican Health Study (MA, USA). They observed that PNC increase was associated with increased BP (both systolic and diastolic). In general, there was a higher positive association among non-smoker and female parts of the population. Moreover, UFP exposure was correlated positively with hsCRP concentration.

Yao et al. [37] examined the data of 2583 participants (without health status discrimination) of the German population-based Cooperative Health Research in the Region of Augsburg (KORA S4) study. Among the 108 studied metabolites, there were nine phosphatidylcholines (PCs) negatively correlated with air pollutants.

### 3.3. Risk of Bias in Studies

**Figure 2:**
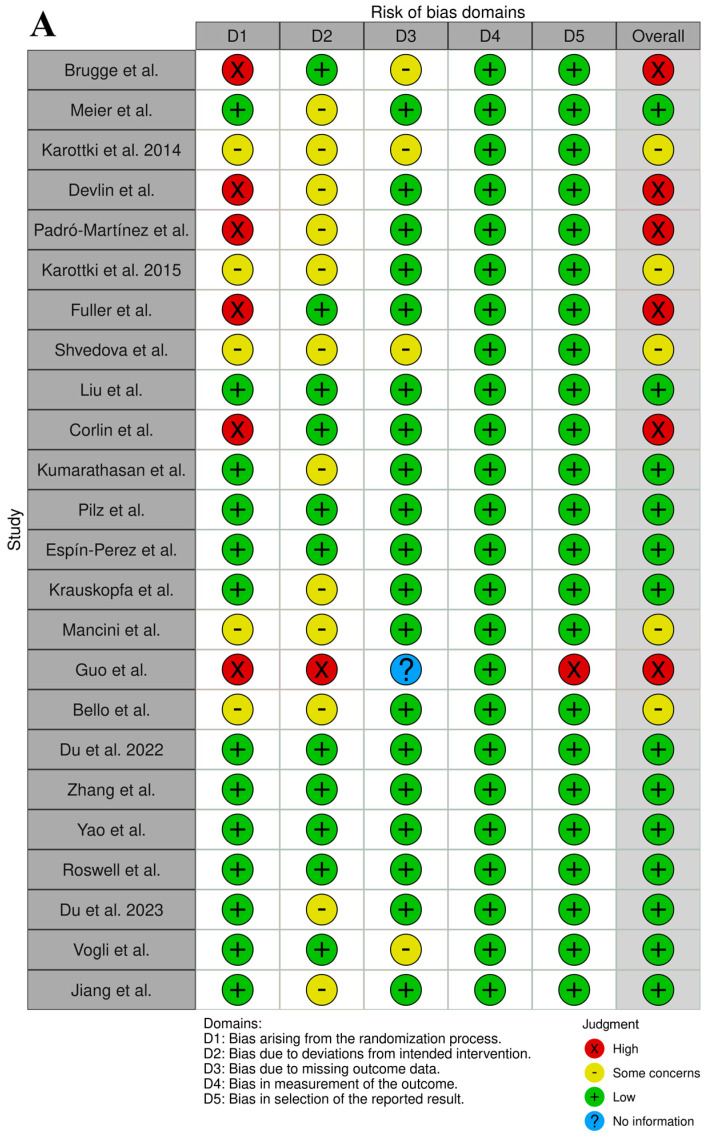

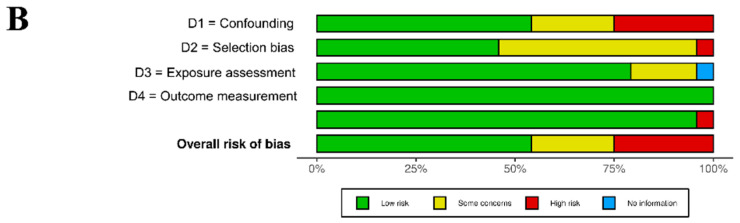
Graphical representation of the RoB assessment results (analysis prepared with open-source software available on https://mcguinlu.shinyapps.io/robvis/ (last entry on 30 December 2024)). (**A**) “Traffic light” graphs of the domain-level evaluations for each study [18,19,20,21,22,23,24,25,26,27,28,29,30,31,32,33,34,35,36,37,38,39,40,41]. (**B**) Weighted bar plots of the distribution of risk-of-bias evaluations of every bias domain.

The analysis of the RoB assignment was made following the WHO instructions, and the analysis is presented in Supplementary Table S3. Moreover, the overall RoB data are presented in Figure 2. The selected records were of satisfactory quality and the association between UFP exposure and imbalance of blood markers of CVDs were properly assigned. The RoB of confounding was high in six studies, which was not considered in the experimental protocol other than for the UFP air pollutants. There were five studies that considered a few air pollutants as possible confounders. The RoB in the selection bias domain (D2) was high in one study with a small number of participants, and there were twelve studies with a low number of participants. There were four studies with some concerns regarding exposure assessment due to measurement methods varying across the range of exposure. However, there was evidence supporting that the exposure measurement is sufficiently similar, and that effect estimates are not strongly biased. In the domains of outcome measurement (D4), all studies were rated as having a low RoB. In the domains of missing data, all studies were rated as low risk, except for the study by Guo et al. [33], in which the risk was high due to missing exposure data without rationale.

### 3.4. Results of Individual Studies on UFP Effects on CVD Blood Marker Imbalance

#### 3.4.1. Short-Term Effects of UFP Exposure on Blood Markers of CVDs

Three records were analyzed for the short-term effects of UFP exposure on the differential expression of RNA. Guo et al. [33] and Shvedova et al. [25] studied total RNA expression in occupational condition environments of the printing (panel study) and carbon nanotube (cross-sectional study) industries, respectively. Du et al. [39] profiled exosomal lncRNA in healthy college students (crossover study). These three studies differ significantly in the study protocols and studied populations (Table 1). Moreover, the population numerosity was low with some inconsistency in the confounding and selection bias domains. Indeed, the overall RoB score was “high” for Guo et al.’s study [33] and there were “some concerns” for Shvedova et al.’s investigation [25]. While the studies by Shvedova et al. and Guo et al. [25,33] examined healthy workers in indoor spaces, the study of Du et al. examined heathy students in outdoor conditions [39]. There are also significant differences in age and gender between these three study protocols.

The study by Guo et al. [33] showed that there is a number of DE total RNAs (Table 2) associated with the immune system (CD9, GAPT, BGP6, HERC6, MMP9, BHLHE40, GBP1, GZMA), metabolism (KMO, ZC3HL5, RAB6B), inflammation (AOAH), heat shock protein family (DNAJA1), myocardial infarction (LGALS2), oncogene (MRAS), mTOR inhibition (NPRL3), cell growth (SGMS2), apoptosis (SOX4), and glutamate signaling in brain (NETO2) function. Those RNAs were also differentially expressed in rats exposed to the same class of UFPs [44].

The study by Shvedova et al. [25] prepared a detailed analysis of dysregulated mRNAs in the blood of workers exposed to a high concentration of UFPs (Table 2). They found a list of DE mRNAs associated with pulmonary inflammation and fibrosis, systemic, cardiovascular (mRNAs associated with “vasodilation of arteries” and “atherosclerotic lesions”), and carcinogenic outcomes (mRNAs associated with “granuloma/formation of granuloma”) upon exposure to UFPs.

There are no similarities between the DE mRNA results obtained by Guo et al. [33] and Shvedova et al. [25], except for LGALS2 mRNA being found differentially expressed in the study by Guo et la. and LGALS3 mRNA being indicated by Shvedova et al. (Table 2) [25].

In the study by Du et al. [39], there were individuated top 20 DE lncRNAs associated with exposure to air pollutants (Table 2). These lncRNAs were involved in biological processes of CVDs.

The differential expression of miRNA as a result of UFP exposure was studied by Shvedova et al. [25] (cross-sectional study), Mancini et al. [32] (panel study), Espin-Perez et al. [30] (crossover study), Krauskopf et al. [31] (crossover study), and Du et al. [35] (crossover study).

The list of specific miRNA sequences found to be common in five different studies is presented in Table 3. There are two miRNA sequences, namely hsa-miR-24-3p and hsa-let-7d-5p, that were found to be differentially expressed after UFP exposure in three studies, even if there are significant differences in the experimental protocols of the five studies. Shvedova et al. and Mancini et al. conducted observational studies [25,32], while the three remaining studies were experimental crossover studies. Participants of all studies were healthy, young or middle age, and both men and women. Only the study of Shvedova et al. looked at indoor UFP exposure effects in working environments [25]. The data collection time after exposure varied among studies or was not defined by the authors (Table 1).

Hsa-miR-24-3p (https://rnacentral.org/rna/URS000059273E/9606, last access date 1 March 2025) is located in extracellular exosomes and in extracellular space. It was reported to be involved in the cell growth involved in cardiac muscle cell development; the miRNA mediation of post-transcriptional gene silencing, gene silencing by inhibition of translation, and mRNA destabilization; the negative regulation of angiogenesis, blood vessel endothelial cell proliferation involved in sprouting angiogenesis, blood vessel endothelial cell migration, voltage-gated sodium channel activity, sodium ion import across plasma membrane, vascular endothelial cell proliferation, protein serine/threonine kinase activity, interferon gamma secretion, osteoblast differentiation, cell migration, cell population proliferation, metalloendopeptidase activity, macrophage migration, cardiac muscle cell apoptotic process, regulation of tumor necrosis factor-mediated signaling pathway, and fibroblast apoptotic process; and the positive regulation of the endothelial cell apoptotic process, vascular-associated smooth muscle cell apoptotic process, reactive oxygen species biosynthetic process, and ERK1 and ERK2 cascade.

Hsa-let-7d-5p (https://rnacentral.org/rna/URS00000A07C1/9606, last access date on: 1 March 2025) is a miRNA (located in extracellular space and vesicles) that is implicated in the development of esophageal squamous cell carcinoma [45]. In a study comparing the expression of various miRNAs in acute myocardial infarction (AMI) patients and normal controls, hsa-let-7d was found to be differentially expressed [45].

The other 11 miRNA sequences were differentially expressed in at least two studies.

There were 10 studies [19,20,21,23,24,28,34,36,38,41] investigating the imbalance of blood markers (different than hsCRP) after short-term exposure to UFPs, and three studies [18,22,40] examining the long-term effects. Table S4 presents summarizing data on inflammation and coagulation blood markers, as well as lipids and other biomarkers dysregulated after UFP exposure, while Table 4 shows data of only blood markers, which were significantly changed after exposure. Most of the blood markers, which significantly change after UFP exposure, are increasing after short-term exposure, namely granulocyte–macrophage colony-stimulating factor, interferon-induced T-cell α chemoattractant, IFN-γ, IL-1α, IL-1β, IL-8, IL-10, lymphocytes, monocytes, fibrinogen, LDL, A2M, adipsin, AGP, haptoglobin, L-selectin, PF4, and ET 1-21. There are two blood markers, which decrease after UFP exposure: HDL and MVF. Regarding leucocytes and IL-6, we can observe that in different studies the results are contrasting. Of note is that there were three independent studies [34,36,41] showing that IL-1β increases after short-term UFP exposure.

Noteworthily, the experimental conditions and data collection protocols differ significantly among the ten selected studies (Table 1). Undoubtedly, variation in the selected populations, gender distribution among participants, age, lifestyle, and eventual comorbidities influenced the final results. For instance, in the studies by Karottki et al. [20,23] the results are changing with different experimental protocols.

#### 3.4.2. Long-Term Effects of UFP Exposure on Blood Markers of CVDs

The studies on the long-term effects of UFP exposure focused mainly on hsCRP. Only three studies [18,22,40] examined a few blood markers. However, the results were contrasting. For instance, in the study by Padrò-Martinez et al. [22] IL-6 decreases after exposure, while according to the data presented by Brugge et al. [18] IL-6 increases after exposure. Such discrepancies could be explained by the heterogeneity among studies due to the lack of common protocols. Each study enrolled a different numerosity population of different age, gender distribution, and health statuses (Table 1). Of note is that the time of sample collection after exposure is significantly different among the selected studies.

### 3.5. Meta-Analysis

**Figure 3:**
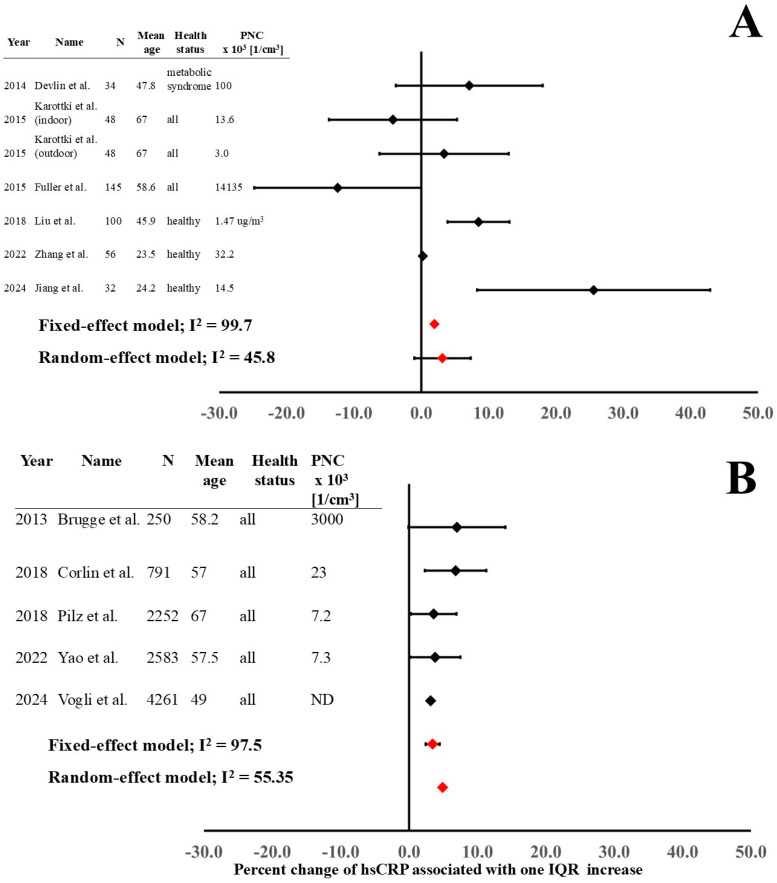
Short-term (**A**) and long-term (**B**) outcome estimates on hsCRP imbalance after UFP exposure [18,20,21,23,24,26,27,29,36,37,40,41].

There were seven studies analyzing the short-term effects of UFP exposure on hsCRP imbalance and five studies investigating the long-term effects, which were used for the meta-analysis. There was one study [19] that was not included in the meta-analysis due to the impossibility of properly extrapolating the required data. The results of the meta-analysis of hsCRP imbalance after UFP exposure are presented in Figure 3.

The results of fixed-effect and random-effect models of both short-term and long-term studies demonstrate a significant increase in hsCRP after exposure (Figure 3). In the short-term studies, the investigations with young and healthy populations [26,36,41] showed a significant increase in hsCRP, while studies with older populations and without health status discrimination presented more heterogeneous results. The low number of studies meant we were unable to carry out the dose–response and time–response analysis.

Significant heterogeneity (expressed as I^2^) can be observed both in short-term and long-term exposure data. The main reasons for high heterogeneity are differences in experimental protocols (Table 1). The enrolled populations (different age, gender distribution, and health status), precision in PNC analysis (personal exposure measurements, fixed measuring points, air quality computational simulations; laboratory-controlled conditions or real-world environments), PNC, time of blood sampling after exposure, and different confounding elements considered in the analysis of effect estimates are significantly determining the heterogeneity. In addition, different study designs (cross-sectional, cohort, panel, and crossover studies) have an effect on the high heterogeneity.

The final-outcome models for the short- and long-term effects on hsCRP were analyzed with both fixed-effect and random-effect models (Figure 3) because of the high heterogeneity.

## 4. Discussion

The study with meta-analysis presented here synthesized the short-term and long-term outcomes of UFP exposure on blood markers of cardiovascular diseases from 24 selected epidemiological studies published in the scientific literature in the last 11 years.

In the short term, we found DE RNA sequences involved in immune, inflammation, atherosclerotic lesion, and vasodilatation of arteries processes, and other lncRNAs previously associated with air pollutant exposure. Moreover, there were a number of miRNA sequences found to be DE in different independent studies. In addition, there were different immune and inflammation blood markers (granulocyte–macrophage colony-stimulating factor, interferon-induced T-cell α chemoattractant, IFN-γ, interleukins-1α, -1β, -6, -8, -10, leukocytes, lymphocytes, and TNF-α) as well as other blood markers (fibrinogen, LDL, A2M, adipsin, AGP, haptoglobin, L-selectin, PF4, ET 1-21, HbA1c, glucose, and insulin) of CVDs increasing immediately after exposure. There were two blood markers, namely HDL and MVF, that decreased after UFP exposure. The meta-analysis of hsCRP imbalance showed that protein concentration is increasing after UFP exposure.

There were a few epidemiological studies investigating the correlation between long-term UFP exposure and different concentrations of CVD blood markers. In two independent studies, IL-6 was individuated as a blood marker influenced by UFP exposure. However, the results were contrasting. The meta-analysis showed clearly a significant increase in hsCRP in populations exposed to UFPs for a long time.

Our findings of the hsCRP analysis in both the short-term and long-term effects remained robust in multiple sensitivity analyses. The heterogeneity in effect estimates across studies could be a result of significantly different study designs in terms of blood sampling time after exposure, type of UFP monitoring, and heterogeneous population enrolled. Moreover, heterogeneity could be partially explained by pre-existing health complications in populations selected for the studies, with greater UFP-associated changes in hsCRP among populations without health complications. In contrast, the PNC was unlikely to be a source of heterogeneity based on our analyses. However, the low number of studies meant we were unable to carry out the proper statistical analysis.

According to Vidale and Campana [46], UFP exposure may lead to the development of CVDs in classic, alternative, or central pathways, with different cardiovascular outcomes depending on the dose and duration of exposure. Short-term exposure on high PNCs led to plaque vulnerability and rapture (classic pathway), thrombosis (alternative pathway), and arrythmias and BP increase (central pathway). Subacute exposure led to atherosclerosis and systemic inflammation (classic pathway), enhanced coagulation (alternative pathway), and HR variability (central pathway). Finally, long-term exposure led to atherosclerosis (classic and alternative pathway) and metabolic syndrome (classic pathway). The classic pathway is articulated by lung production of proinflammatory mediators and their release in the systemic circulation. In the alternative pathway, compounds penetrate the circulation directly from the lung. In a central pathway, alveolar receptors directly stimulate the autonomic nervous system.

Insulin resistance, hypertension, endothelial dysfunction, oxidative stress, and dyslipidemia are the main pre-conditions of CVDs, namely myocardial infarction and ischemia, arrhythmias, coronary thrombosis, atherosclerosis, and ventricular enlargement [47]. Biomarkers of cardiac diseases [48] are molecules secreted in the blood by the injured or strained cardiac tissues. There is a list of well-known protein, lipid, genome-based, carbohydrate-based, nucleic acid-derived, and pre-disease biological biomarkers associated with CVDs [47].

Anthropogenic UFPs generated in the combustion processes have a porous structure increasing the surface-to-volume ratio of the particles and often contain toxic metal ions and organic species. Copper, iron, and manganese are only a few of the metal ions with redox potential and can produce reactive oxygen species (ROS), leading to oxidative stress and systemic inflammation development [7,49]. Inhaled nanoparticles may translocate to the brain through axonal transport from the olfactory mucosa into the olfactory bulb and/or the blood circulation after alveolar deposition [50]. Different studies have demonstrated that UFPs may translocate from the lungs to the circulation due to their low diameter and induce lung oxidative stress with a resultant increase in lung epithelial permeability. UFPs may cause ROS generation in endothelial cells by the activation of NADPH oxidase. UFP-induced ROS lead to activation of MAPKs through induced phosphorylation of p38 and ERK1/2 MAPKs that may further result in endothelial dysfunction through production of cytokines such as IL-6 [51].

In the last decade, numerous studies have focused on gene expression profiling as biomarkers of cardiovascular diseases. Recently, Musunuru et al. [52] reviewed the latest advances in genome expression in CVDs (i.e., coronary artery disease, myocardial infarction, ischemic cardiomyopathy, sudden cardiac death, and stroke). In the systematic review presented here, two independent studies presented a number of mRNA sequences, which are differentially expressed upon UFP exposure. In both studies, DE mRNAs were associated mostly with inflammatory and immune processes. The comparison of two studies (Table 2) showed that LGALS2 and LGALS3 were differentially expressed in both studies [25,33]. LGALS genes encode galectin proteins, a class of proteins that bind specifically to β-galactoside sugars. There are 15 different types of galectin proteins with specific functions in immune system, inflammation, wound healing, and carcinogenesis processes. Recent studies showed that galectins act both by relieving ischemia and accelerating atherosclerosis. Moreover, galectins play a role in the development of myocarditis by their influence on inflammatory processes. In addition, some findings showed that galectins act as a biomarker for the severity of myocardial ischemia and heart failure. The recent exhausting reviews on galectins’ role in CVDs can be found elsewhere [53,54].

Long non-coding RNAs (long ncRNAs, lncRNA) are transcripts more than 200 nucleotides long that are not translated into protein. LncRNAs can regulate cellular biological processes through numerous molecular processes. The high number of lncRNAs in the cardiovascular system suggest their importance in cardiovascular physiology and pathology. LncRNAs were suggested to be used in the diagnosis and monitoring of heart failure by Zhang et al. [55], and to diagnose ST-elevation myocardial infarction [56]. Among the top 20 DE lncRNAs described by Du et al. [39], there is metastasis-associated lung adenocarcinoma transcript 1 (MALAT1). It is also secreted as a cfRNA, and exosomal MALAT1 was found to affect the formation of “neutrophil extracellular traps” (=decondensed extracellular neutrophil chromatin) in a mouse model of atherosclerosis [57]. LncRNA activated by DNA damage (lncNORAD) regulates inflammation, lipid levels, and atherosclerosis in various cardiovascular diseases. LncNORAD was higher in coronary heart disease (CDH) patients than in controls [58]. Moreover, lncNORAD was positively correlated with hsCRP, TNF-α, IL-6, IL-8, and IL-17A. Furthermore, lncNORAD was positively associated with total cholesterol and LDL cholesterol, whereas it was not related to triglyceride and HDL cholesterol. Fan et al. [59] showed that lncNORAD was overexpressed in the serum of carotid artery stenosis patients, and it was associated with patients’ hypertension, total cholesterol, LDL cholesterol levels, and stenosis degree. Small nucleolar RNA host gene 6 (SNHG6) expression increased in hypoxia/reoxygenation (H/R)-challenged cardiomyocytes [60]. LncRNA H19 is known to be involved in the pathophysiological processes of CVDs. Recent reviews by Li et al. [61] and Busscher et al. [62] exhaustively synthesized the current knowledge on lnc H19’s role in cardiovascular biology and diseases (atherosclerosis, hypertension, coronary artery disease (CAD), myocardial infarction (MI), myocardial ischemic-reperfusion injury, pulmonary arterial hypertension, heart failure, aortic aneurysm, and aortic dissection). Kot et al. [63] showed that the low expression of LRRC75A-AS1 was correlated with enhanced inflammatory response and thus increased plasma hsCRP, IL-6, and TNF-α. LncRNA Tumor Protein Translationally Controlled 1 (TPT1) Antisense RNA 1 (TPT1-AS1) was found to be imbalanced in different diseases and correlated with patient prognosis. It was demonstrated that TPT1-AS1 modulates different biological processes, among which are cell proliferation, apoptosis, autophagy, invasion, migration, radiosensitivity, chemosensitivity, stemness, and extracellular matrix synthesis. Recently, Li et al. [64] reviewed the role of TPT1-AS1 in human diseases, focusing on its expression, relevant clinical characteristics, molecular mechanisms, biological functions, and subsequent clinical applications. In 2023, Zhang and Chen [55] presented bioinformatic gene analysis for potential biomarkers and therapeutic targets of heart fibrillation (HF) and stroke. They found that 21 co-expressed DE genes and two co-expressed lncRNAs (MALAT1 and GABPB1-AS1) are associated with HF-related stroke. Wang et al. [65] presented data supporting the theory that lncRNA LUCAT1 offers protection against human coronary artery endothelial cellular oxidative stress injury by modulating the hsa-miR-6776-5p/LRRC25 axis and activating autophagy flux. According to the authors, during the progression of human coronary atherosclerosis there is a dynamic disruption of the autophagy landscape, which could be protected by the lncRNA LUCAT1/hsa-miR-6776-5p/LRRC25-driven mechanism of autophagy activation. In the case–control study by Wang et al. [65] low LUCAT1 expression was associated with the poor prognosis of patients with chronic heart failure (CHF) and was an independent prognostic factor for their survival.

MiRNAs are small, single-stranded, non-coding RNA molecules composed of 21–23 nucleotides. MiRNAs are involved in cell-to-cell communication as well as epithelial–mesenchymal transition. In addition, they are correlated with myocyte hypertrophy and left ventricular remodeling [48], and regulation of cardiomyocyte apoptosis and oxidative stress processes [66]. MiRNA sequences were proposed to be used in the early diagnosis of acute heart transplant rejection [67], risk of stroke [68], pre-eclampsia, pulmonary hypertension, and vasculitis [69]. Our analysis showed that two miRNA sequences, namely miRNA-24-3p/miRNA-24-5p and hsa-let-7d-5p/hsa-let-7d-3p, were found in three independent studies [25,32]. In the recent study by Lin et al. [70], miRNA-142-3p levels were significantly upregulated in a cohort study of 30 clopidogrel-resistant patients with respect to the 30 healthy volunteers control group. In 2022, Jia et al. [71] showed that LINC00460 (lncRNA) regulates the proliferative ability of endothelial cells (ECs) and thus the occurrence and development of coronary atherosclerotic diseases by targeting miRNA-24-3p. Aroca-Esteban et al. [72] showed that let-7d-5p is increased in carotid plaques from overweight patients with carotid atherosclerosis. Additionally, in extracellular vesicles (EVs) isolated from plasma of patients with carotid atherosclerosis, the let-7d-5p levels were higher than in control subjects. The differences were significantly marked in overweight patients. In 2021, Lightbody et al. [73] established that exposure to lipoproteins, including acetylated LDL, induced macrophage expression of microRNA hsa-let-7d-5p. Moreover, hsa-let-7d-5p inhibition significantly increased endogenous biosynthesis of cholesterol and cholesteryl ester pools in macrophage “foam” cells, without altering the cholesterol efflux pathway or esterification of exogenous radiolabeled oleate. Vartak et al. [74] showed that let-7d-5p overexpression significantly attenuated TNF-α-induced upregulation of *IL-6*, *ICAM1*, *VCAM1*, *CCL2*, *CD68*, and *MYOCD* gene expression in target human aortic smooth muscle cells. In 2019, Recamonde-Mendoza [75] showed that different transcription factors (TFs) and miRNAs (among which are hsa-let-7i-5p and hsa-let-7e-5p) were central nodes in the network and point to potential active regulatory pathways in cardiac hypertrophy (CH).

Lipid metabolism, glucose homeostasis, vascular integrity, endothelial cell function, and cytokine-activated inflammation are only a few pathways influenced by miRNA regulation [76]. Numerous studies showed that a miRNA panel’s combined utility increased the predictive value of classic Framingham risk models, but any single miRNA imparted a clinically relevant reduction in acute myocardial infarction (MI) risk [77].

Investigation of peptide markers of CVDs in blood has been a leading topic of CVD early diagnosis, as well as prognosis. There is a number of reviews synthesizing knowledge of well-established as well as novel and promising peptide biomarkers of different CVDs [78,79,80]. Here, only a few important mechanisms of CVD blood markers are discussed. For instance, TNF-α was found to be elevated in chronic heart failure (HF) [81] and newly diagnosed HF patients [82]. In addition, TNF-α was proposed as a predictor of death in advanced HF patients [83]. IL-6 regulates cardiomyocyte hypertrophy and apoptosis [84]. Cardiac IL-6 expression increases in advanced HF [85]. In particular, high IL-6 levels are present before HF diagnosis [86] and suggest that IL-6 could be used in recognition between the decompensated and compensated phases of HF [87]. The role of different cytokines in CVDs has been recently reviewed by Haybar et al. [88]. IFN-γ is considered a mediator in atherosclerosis [89,90]. The main hematopoietic parameters [91] and blood lipid profiles [92,93,94] change significantly before different CVDs are diagnosed. However, they are not specific.

Fibrinogen is an important acute-phase protein in coagulation cascade [95]. It is a significant and independent CVD risk factor associated with other risk factors and genetic variants. Moreover, fibrinogen has emerged as an essential novel predictor of CVD risk [96,97]. Fibrinogen’s role in CVDs has been reviewed elsewhere [98,99,100].

The serum amyloid A (SAA) is an apolipoprotein, which are associated with lipoproteins (namely HDL). SAA1 and SAA2 are released into blood stimulated by IL-6 in response to infection, inflammation, injury, or stress. Among the different binding-phase proteins examined as predictors of CVD progression, SAA has shown to be a promising candidate as a CVD biomarker [101].

Recently, Castro et al. [102] presented a narrative review summarizing the latest advances in the use of hsCRP detection in CVDs. HsCRP production is stimulated by proinflammatory cytokines. Since hsCRP is involved in the formation of atherosclerotic plaque, its high concentration is considered a CVD risk factor when used in clinical practice as a biomarker. HsCRP is synthesized mainly by the liver upon response to IL-1 and IL-6 due to tissue infection or damage. Locally it is secreted by endothelial cells, atheroma plaque, muscle cells of the arterial coronary wall, and adipocytes [103]. Of note is that its concentration is positively correlated with cholesterol in patients with coronary disease. However, hsCRP concentration is not affected by diet or circadian variations. HsCRP synthesis initiates 4–6 h after inflammation stimulus and duplicates every 8 h, thus reaching a peak 2 days after the stimulus. The hsCRP half-time lasts up to 19 h. However, it can be days until it returns to basal levels [104]. Even if highly sensitive, hsCRP is an unspecific inflammation marker associated with not only CVDs but also other inflammatory diseases in different tissues. Even if the feasibility and cost of hsCRP quantitative analysis are low, the lack of specificity, low stability, and lengthy detection period of hsCRP are considered the main drawbacks in the use of hsCRP as a biomarker of CVDs. Nevertheless, hsCRP is an unspecific inflammation marker. The blood hsCRP increase does not indicate a specific tissue, which is under the inflammation state. For instance, inflammatory bowel disease [105], psoriasis [106], and rheumatoid arthritis [107] are only a few of the inflammatory diseases characterized by the increase in hsCRP.

Nuclei acids, namely mRNA, lncRNA, and miRNA, released directly into circulation or packaged into exosomes, play important roles in many biological processes. The review presented here showed that combining RNA expression data and the concentration of relative proteins or lipids, thus constructing an RNA–protein–lipid-based transcriptional network, could be helpful in understanding the pathomechanisms of CVDs, which are activated by UFP exposure. Moreover, the entire nucleic acid–protein–lipid profiles, rather than single biomarkers, could be used in an effective prediction and/or diagnosis of CVDs in a highly selective way.

The analysis presented here of different blood markers of CVDs showed that there is a heterogeneity among study protocols. The future studies on the health effects of UFP exposure should consider the unified protocols, which permit better comparison of different studies. However, the proper guidelines on common protocols are still missing.

Despite the high heterogeneity of the selected studies, there is a general trend in the different blood marker increases after UFP exposure both in the short term and long term. Fragile populations are particularly at risk and should be covered by additional prevention measures by the health care system. Moreover, particular attention should be paid to indoor air quality, with proper measures against UFP generation during combustion processes (e.g., meal frying and grilling) and proper preventive education of UFP hazards.

## 5. Conclusions

In conclusion, our study supports an association between short-term and long-term exposure to UFPs and imbalance of selected blood markers. In particular, there is a significant increase in hsCRP after both short-term and long-term exposure. As expected, the increased inflammatory processes due to UFP exposure led to increases in IL-6, IL-1β, and other inflammatory markers. Nucleic acid differential expression analysis is an emerging tool in the study of air pollutants’ influence on CVD development and progression. MiR-24-3p and let-7d-5p are two miRNAs sequences, which were DE in different populations exposed in the short term to UFPs. Moreover, LGALS mRNA and lnc-NORAD were two nucleic acids for which expression was dysregulated in populations exposed to UFPs for a short time. Our findings highlight the possibility to define and quantify more precisely the CVD risk factors in air-polluted areas by using the combination of BP, HRV measures, and selected blood biomarker analysis. The analysis presented here underlines the heterogeneity among the selected studies, which limits the statistical analysis. Future studies should pay more attention to study protocols, with the aim of obtaining more homogeneous results.

## 6. Strengths and Limitations

The key strength of the study presented here is that it provides for the first time a quantitative analysis of the UFP effects on hsCRP concentration, a key blood marker of inflammation related to CVDs. We also acknowledge the limitation that there is a heterogeneity among the studies selected for meta-analysis. However, potential sources of heterogeneity were indicated. Further studies are still needed to disentangle the independent UFP effects from co-pollutants and other confounders. More attention should be paid to experimental protocols. The use of common study models (in terms of population enrollment, choice of confounders and statistical analysis, and type of UFP monitoring protocols) should be helpful in reducing the heterogeneity of results. Personal exposure assessment quantifies more accurately the real UFP exposure; thus, more research on personal UFP exposure should be warranted also in long-term studies. Considering the limitations of using single-marker analysis or a single physical exam, combined BP/HRV and selected blood marker analysis should be used more frequently in air pollutant studies.

## Figures and Tables

**Table 1:** Main data synthesizing records selected for the systematic review. ND = data not delivered.

No	Year	First Author	Study Design	Location	Time Outcome	Study Time Range	Population	Exposure Location	Experiment	Exposure Assignment	Size Range (nm)	Mean ± SD (Range) (×10^3^ particles/cm^3^)	Pollutants/Risk Factors	Exposure Quantification	Outcome	Time After Exposure of Outcome Measure
1	2013	Brugge et al. [18]	cross-sectional	Boston, MA, USA	long-term	7.2009–6.2011	260 adults (mean age 58.2; 58% women), no health status discrimination	outdoor	real environment	mobile monitoring of particle number concentration	ND	3000	only UFPs	continuous	high-sensitivity C-reactive protein (hsCRP), interleukin-6 (IL-6), tumor necrosis factor alpha receptor II (TNF-RII), and fibrinogen	ND
2	2014	Meier et al. [19]	panel study	Western Switzerland	short-term	5.2010 and 2.2012	18 participants (healthy male highway maintenance workers aged 31–59 years)	indoor and outdoor	occupational environment	personal monitoring	<100 nm	75.699 ± 81.761	PM_2.5_, noise, and gaseous co-pollutants (CO, NO_2_, O_3_), temperature, humidity	continuous	blood pressure (BP), IL-6, TNF-α, hsCRP, serum amyloid A (SAA), lung function, electrocardiogram (ECG), heart rate variability (HRV), fractional exhaled nitric oxide (FeNO)	exposure to PM_2.5_, UFP, noise, and gaseous co-pollutants were assessed during five nonconsecutive work shifts; to control post-work-shift exposure, personal PM_2.5_ real-time and noise exposure measurements were continued after the end of work (around 1700 h) until the next morning
3	2014	Karottki et al. [20]	cross-sectional	Copenhagen, Denmark	short-term	10.2011–2.2012	78 healthy, middle-aged participants (mean age data ND); 33 women and 45 men	indoor and outdoor	real environment	indoor PNC monitored for 48 h with Philips NanoTracer1000; outdoor PNC measured with ambient air pollution data by Aarhus University (Danish Air Quality Monitoring Programme)	10–300	indoor: 12.4 (median); outdoor: 3.9 (median)	indoor bacteria, endotoxins, and fungi; PM_10_ and PM_2.5_	continuous	MVF, HbA1c, hsCRP, leukocytes, monocytes, neutrophils, CD31, CD62L, CD11b, CD49d, FEV1/FVC, eosinophils, lymphocytes	microvascular function (MVF); blood pressure (BP); forced expiratory volume in the first second (FEV1) and forced vital capacity (FVC)
4	2014	Devlin et al. [21]	crossover randomized	Campus of the University of North Carolina	short-term	data not delivered	34 middle-aged participants with metabolic syndrome (13 male and 21 female), mean age 47.8	exposure chamber	laboratory exposure chamber	monitored in real-time using a TSI 3022A CPC	20–250	100	only UFPs	2 h	holter ECG (SDNN, PNN50; HF, LF, premature atrial contractions (PACs) and premature ventricular contractions (PVCs)), brachial artery diameter (BAD), endothelium-dependent flow-mediated dilatation (FMD), and nitric oxide-mediated dilatation (NMD), images of the right brachial artery (BAD1) were captured at the end-diastole	participants were exposed two times for 2 h (clean air and concentrated ambient ultrafine particles)
5	2015	Padró-Martínez et al. [22]	double-blind crossover trial	Somerville, MA, USA	long-term	2. 2011–11. 2012	20 participants (17 women); mean age 53.9	indoor	real environment	stationary particle counter	7–3000	4.8	only UFPs	continuous	hsCRP, IL-6, TNF-RII, and fibrinogen; BP	first day: before HEPA/sham filtration was started; twenty-first day: 1–2 h before the filters were changed; forty-second day: before the end of this study
6	2015	Karottki et al. [23]	crossover	Copenhagen, Denmark	short-term	11.2010–5.2011	48 elderly subjects (22 men and 26 women); 67 ± 7 (mean ± SD) years old, no health status discrimination	indoor and outdoor	real environment	Danish Air Quality Monitoring Programme and custom-built Differential Mobility Particle Sizer (outdoor); PNC was monitored for about 48 h with Philips NanoTracer1000 (indoor)	10–300	13.6 (indoor); 3.0 (outdoor)	PM_2.5_	continuous	bacteria, endotoxins, fungi, serine protease, MVF, leucocytes, lymphocytes, monocytes, granulocytes, CD31, CD62, CD11b, FEV1/FVC, CC16, SPD	hours–days
7	2015	Fuller et al. [24]	cross-sectional	Boston, Massachusetts (United States)	short-term	8. 2009–10. 2010	125 participants, 58.6 mean age, 99 female and 43 male, no health status discrimination	outdoor	real environment	ambient monitoring station	<100 nm	14,135 (interquartile range [IQR]: 7314–19,964)	only UFPs	continuous	IL-6, hs-CRP, and fibrinogen	between 1 and 28 days
8	2016	Shvedova et al. [25]	cross-sectional	Kazan, Russia	short-term	data not delivered	15 healthy workers; gender data ND	indoor	working environment	pump collecting particles	<100 nm	14.42 ± 3.8 [µg/m^3^]	elemental carbon (EC)	continuous	miRNA, lncRNA, mRNA	ND
9	2018	Liu et al. [26]	panel study	Northern Taiwan	short-term	1. 2014–8. 2017	100 healthy adults (non-smoking, age range of 20–64 years; mean age 45.9 ± 7.2); 50% women	indoor	real environment	stationary particle counter	50–100	1.47 ± 0.88 [µg/m^3^]	PM_10_, PM_2.5_, NO_2_, O_3_, temperature, and relative humidity	continuous	SBP, DBP, FEV1, hsCRP	each participant was repeatedly interviewed and examined three times occurring at one-month intervals
10	2018	Corlin et al. [27]	longitudinal	Massachusetts, USA	long-term	2004–2015	791 adults (69% women) participating in the longitudinal Boston Puerto Rican Health Study; mean age 57; no health status discrimination	outdoor	real environment	residential annual average UFP exposure assigned with model accounting for spatial and temporal trends (data from mobile and stationary platforms, meteorological data, and distances from specific roadways and bus routes)	<100	23 (3.4)	only UFPs	continuous	systolic blood pressure, diastolic blood pressure, hsCRP, particle inhalation rate (PIR)	participants were visited up to three times over approximately six years (visit one between 2004 and 2009, visit two between 2006 and 2011, and visit three between 2011 and 2015); the mean time between visit one and visit two was 2.2 years while the mean time between visit two and visit three was 4.1 years
11	2018	Kumarathasan et al. [28]	crossover randomized	Sault Ste. Marie, Ontario, Canada	short-term	summer 2010	52 healthy participants (aged 18–34), median age 23; 28 female and 24 male	outdoor	real environment	fixed-site ambient air quality monitor	10–1000	14.830 (13.604; 16.057)	SO2, NO_2_, NOx, O_3_, temperature and relative humidity, air pressure	continuous (hourly between 8 h and 18 h)	salivary ET-11-21, ET-11-31, ET-3, and BET-1; hsCRP, haptoglobin, fibrinogen, platelet factor (PF4), adiponectin, von Willebrand Factor (vWF), α2-macroglobulin (A2M), α-acid glycoprotein (AGP), serum amyloid protein (SAP), L-selectin, and cytokines [interleukins (IL-1, -2, -4, -5, -6, -7, -8, -10, -12, -13), tumor necrosis factor (TNF-α), granulocyte–macrophage colony-stimulating factor (GMCSF), and interferon gamma (IFN-γ)], BET-1	at the end of the week
12	2018	Pilz et al. [29]	cross-sectional	Region of Augsburg, Germany	long-term	3.2014–4.2015	2252 participants, mean age 60.3 ± 12.3; no health status discrimination; male 1091 (48.4%)	outdoor	real environment	framework of the ULTRA 3 project modeling; NanoScan SMPS Nanoparticle Sizer	10–420	7.2	PM_10_, PM_2.5_, NO_2_ or NOx, O_3_; traffic noise	continuous	hsCRP	ND
13	2018	Espín-Perez et al. [30]	crossover	Barcelona, Spain; London, United Kingdom	short-term	ND	59 (London) and 30 (Barcelona) healthy volunteers, mean age data not delivered; 50% female	outdoor	real environment	real-time personal portable monitors	<100	Barcelona: 46.481 ± 21.027; London: 166.667 ± 28.759	PM_10_, PM_2.5_, NO_2_, NO_x_, BC, CO, CO_2_	continuous	miRNA	few hours
14	2018	Krauskopf et al. [31]	crossover	London, United Kingdom	short-term	ND	24 volunteers (12 male and 12 female), mean age 65.1 (7.7); healthy, COPD, and IHD patients	outdoor	real environment	real-time personal condensation particle	100 nm	Hyde Park: 5.975 (CI 4.815–7.133); Oxford Street: 28.656 (CI 25.803–31.509)	BC, NO_2_	continuous (2 h)	miRNA	few hours
15	2020	Mancini et al. [32]	panel study	Switzerland (Basel), United Kingdom (Norwich), Italy (Turin), and The Netherlands (Utrecht)	short-term	12, 2013–2, 2015	143 healthy subjects (>40 participants per country), mean age data ND; 86 women and 56 men	outdoor	real environment	real-time personal portable monitors	10–300	6.318 (0.785–22.536)	PM_2.5_	continuous	total RNA	three sessions at different seasons within 12 months
16	2020	Guo et al. [33]	panel study	Singapore	short-term	NA	11 health workers, aged > 21 years old, mean age data ND; controls from both companies were female, and all subjects in Company 2 were female (numerical data ND)	indoor	working environment	ND	ND	ND	only UFPs	ND	total RNA, protein expression of sICAM	four time points (TPs) over a 2-week period: the first Monday, the first Friday, the second Monday, and the second Friday
17	2021	Bello et al. [34]	cross-sectional	Singapore	short-term	2018–2019	19 healthy workers, mean age 36.2 ± 12.1; 9 female and 10 male	indoor	working environment	personal monitoring	10–420	1.680–49.900 (week means)	PM_2.5_	continuous (at least 4 h a day)	forced expiratory volume in the first second (FEV1), forced vital capacity (FVC), and FEV1/FVC ratio greater than 0.8; IL-1β; IL-6; IL-8; IL-19; eotaxin; fractalkine; GCSF; IFN-gamma; GM-CSF; TNF-α; MCP1; EGF; IL-1α; VEGF	between 1 and 3 days
18	2022	Du et al. [35]	crossover randomized	Shanghai, China	short-term	10–12, 2019	56 healthy participants (25 males and 31 females); mean age data ND	outdoor	real environment	real-time personal portable monitors	10–100	road session: 33.83 (7.24); park session: 15.61 (4.76)	PM_2.5_, BC, NO_2_, and CO_2_	continuous	exosomal miRNA	ND
19	2022	Zhang et al. [36]	crossover	Shanghai, China	short-term	10–12.2019	56 healthy participants (25 males and 31 females); mean age 23.5 ± 2.4	outdoor	real environment	real-time personal exposure	ND	park: 13.4 (11.7, 17.0); road: 32.3 (29.4, 39.7)	BC, NO_2_, CO, PM_2.5_, noise, temperature, relative humidity	continuous	28 targeted biomarkers	1 h
20	2022	Yao et al. [37]	longitudinal panel study	Region of Augsburg, Germany	long-term	1999–2013	2583 whole population without health status discrimination; mean age 57.5 ± 13.3; 1240 male	outdoor	real environment	estimated using land-use regression (LUR) models	≤100 nm	7.3 ± 1.8	PM10, PM_coarse_, PM_2.5_, NO_2_, NO_x_, O_3_	ND	hsCRP, metabolites (amino acids, phosphatidylcholines, sphinogmyelins, acylcarnitines, lysophosphatidylcholines, hexoses)	8 h
21	2023	Roswall et al. [38]	cohort	Denmark	short-term	2015–2019	32,851 adult Danes taking part of the Diet, Cancer and Health—Next Generations cohort, mean age 42.5 ± 12.8; 59.1% female	outdoor	real environment	AirGIS modeling system	ND	8.539 ± 2.354	PM_2.5_, EC, NO_2_, noise, intensity of traffic, along with emission factors, meteorology, and street and building configurations	continuous	high-density lipoprotein (HDL), non-high-density lipoprotein (non-HDL), systolic and diastolic blood pressure	between 24 h and 90 days before blood sampling
22	2023	Du et al. [39]	crossover randomized	Shanghai, China	short-term	10–12, 2020	56 healthy participants (31 females and 25 males) mean age data ND	outdoor	real environment	real-time personal portable monitors	10–101	high exposure: 31.61 (24.56−49.89): low exposure: 14.78 (8.12−24.46)	PM_2.5_, BC, CO, NO_2_	continuous	exosomal lncRNA	ND
23	2024	Vogli et al. [40]	cross-sectional	Region of Augsburg, Germany	long-term	1999–2001	4261 participants, aged 25–75 years, mean age 49.0 ± 13.9; 50.5% female; mean age in older subsample 64.0 ± 5.4 and 48.1% female; no health status discrimination	outdoor	real environment	annual average concentrations estimated by land-use regression models and assigned to participants’ home addresses	<100		PM_10_, PM_2.5_, O_3_, NO_2_, NO_x_	continuous	fibrinogen, hs-CRP, SAA, adiponectin, IL-6	ND
24	2024	Jiang et al. [41]	longitudinal panel study	Shanghai, China	short-term	10. 2020–11. 2021	32 participants (15 male, 17 female), 24.2 ± 2.7 mean age, healthy non-smoking	indoor and outdoor	real environment	fixed-site monitors; UFP: Scanning Mobility Particle Sizer (SMPS, TSI Corporation, Washington, DC, USA) and NanoTracer XP	<100 nm	0–3 h: 14.520 (6.153); 4–6 h: 13.991 (5.054); 7–12 h: 10.236 (3.064); 13–24 h: 12.461 (4.612); 25–48 h: 12.305 (4.612)	PM_2.5_, NO_2_, CO, or O_3_	continuous	hs-CRP, TNF-α, interferon-γ (IFN-γ), IL-6, glucose, insulin, total cholesterol, triglyceride, high-density lipoprotein cholesterol (HDL), and low-density lipoprotein cholesterol (LDL)	systemic inflammation (hsCRP, TNF-α, IFN-γ, IL-1β, IL-6): 0–3 h; IL-8: 4–6 h; blood glucose: 0–3 h; blood lipid: DHL 13–24 h, LDL 7–12 h; ApoA-I 25–48 h; ApoB 25–48 h

**Table 2:** Nucleic acids differentially expressed upon UFP exposure and involved in signaling, immune, and inflammation responses.

Nucleic Acid	Concordant DE Genes in Blood Samples of Occupational Workers Exposed to UFP Emissions and PEPs Exposed to Rat Blood by Guo et al. [33]	mRNAs in the Blood of MWCNT High-Exposure Workers Associated with Various Pulmonary and Systemic Outcomes (Obtained with Ingenuity Pathway Analysis) by Shvedova et al. [25]
total RNA	CD9, DNAJA1, GAPT, GBP6, HERC6, KMO, LGALS2, MMP9, MRAS, NPRL3, SGMS2, SOX4, ZC3HL5, AOAH, BHLHE40, GBP1, GZMA, NETO2, RAB6B	Lung inflammation and/or fibrosis: DNRA, PTGIR, PTGS2, IL-6, SPHK1, FGFR1, RETNLB, PLG, CSF2, VTN, CXCL12, EGFR, PPARD, TNFRSF25, CHRNG, ACKR1, FCGR2B, CCR5, TNSF4, HRH1, ST3GAL3, HMGCR, IMPDH1, CDH13, IKBKE, LDLR, BSG, CALCA, MARK2, RORA, LTA, TFPI, LIPA, VEGFA, TRPV4, GATA3, MAPK3, POMC, CD276, CD44, STAT1, GSS, HLA-DRA, IL-11RA, RLN2/3, CD3D, CD46, TSLP, FGFR3, LGALS3, LIF, TLN1, CHRNE, SERPINB1, ANXA1, ADORA1, IMPDH2, PRDX, E2F2, CASP1, CYLD, NRTN, C3AR1 Granuloma: IL-6, LTA, TREM2, HGF, VEGFA, CD44, STAT1, BRAF Immunosuppression: TNFSF4, Pka Bronchoalveloar adenoma: TP63, TNK1, TP73, BRAF, XPA, HTAT1P2, NUDT1 Bronchoalveolar adenoarcinoma: EGFR, TNRC6B Formation of lung tumors: PTGS2, PTN, Integrin, Jnk, S100A10, Cdk, FHIT, ATP synthase, RARB Goblet cell metaplasia: HRH1, ADORA1, LGALS3 Goblet cell hyperplasia: IKBKE, RORA, GATA3, CDH13, TSLP Systemic inflammation: OLR1, LGALS3 Atherosclerotic lesions: GSTT1, LTA, GPX8, FUT4, CCR5, PLG, MMP19, BMP7, VEGFA, CSF2, LDLR, TNFSF4, FUT7, CXCR1, CD44, CCL3L3, FABP4, HMOX2, CD3, GRK4, MIF, LDL Vasodilation of arteries: VEGFA, MFAP5, NOX1, PIK3R1/R3, PLG, LDLR, CALCA, ERK, PLCD3, HMOX2, GUCY1A3, PDGFB, SOD1, RLN2/3, PLCL2, PTPN1, CELA1
Nucleic acid	Top 20 Differentially Expressed lncRNAs Associated with Air Pollution Exposure by Du et al. [39]	
lncRNA	AC093503.2, NORAD, SNHG6, MALAT1, AL138963.3, H19, AC103691.1, LINC01871, LRRC75A-AS1, AP001189.1 LINC02280, TPT1-AS1, BAALC-AS1, AL161457-2, GABPB1-AS1, LINC00632, MCM3AP-AS1, LUCAT1, AC245452.1, AL031595.3	

**Table 3:** List of miRNA sequences DE after UFP exposure in five independent studies.

N.	Shvedova et al. [25]	Mancini et al. [32]	Espín-Perez et al. [30]	Krauskopfa et al. [31]	Du et al. [35]
1	hsa-miR-24-3p	hsa-miR-24-3p			hsa-miR-24-5p
2	hsa-let-7d-5p	hsa-let-7d-5p			hsa-let-7d-3p
3	hsa-miR-425-5p	hsa-miR-425-5p			
4	hsa-miR-505-3p	hsa-miR-505-3p			
5	hsa-miR-16-5p		hsa-miR-16-5p		
6	hsa-miR-197-3p		hsa-miR-197-3p		
7	hsa-miR-29a-3p		hsa-miR-29a-3p		
8	hsa-miR-15a-5p		hsa-miR-15a-5p		
9	hsa-miR-92a-3p		hsa-miR-92a-3p		
10	hsa-miR-133a-3p			hsa-miR-133a-3p	
11	hsa-miR-193b-3p			hsa-miR-193b-3p	
12	hsa-miR-433-3p			hsa-miR-433-3p	
13	hsa-miR-145-5p			hsa-miR-145-5p	

**Table 4:** Blood markers that were significantly imbalanced after UFP exposure.

N.	Study	Name of Marker	Up/Down	Time of Measure After UFP Exposure
1	Zhang et al. [36]	Granulocyte–macrophage colony-stimulating factor (pg/mL)	↑	1 h
2	Zhang et al. [36]	Interferon-induced T-cell alpha chemoattractant (pg/mL)	↑	1 h
3	Jiang et al. [41]	IFN-γ (pg/mL)	↑	0–3 h
4	Bello et al. [34]	IL-1α	↑	two randomly selected consecutive weeks: Monday AM and Friday PM on both Week 1 and Week 2 during 2018–2021
5	Zhang et al. [36]	IL-1β (pg/mL)	↑	1 h
6	Bello et al. [34]	IL-1β	↑	two randomly selected consecutive weeks: Monday AM and Friday PM on both Week 1 and Week 2 during 2018–2019
7	Jiang et al. [41]	IL-1β (pg/mL)	↑	0–3 h
8	Padró-Martínez et al. * [22]	IL-6	↓	10^4^ particles/cm^3^ increase in PNC moving average (MA) (14- or 21-day MA) adjusted for baseline blood biomarker concentrations
9	Jiang et al. [41]	IL-6 (pg/mL)	↑	0–3 h
10	Brugge et al. * [18]	IL-6	↑	in the morning in the study areas
11	Brugge et al. * [18]	IL-6	↑	
12	Jiang et al. [41]	IL-8 (pg/mL)	↑	0–3 h
13	Zhang et al. [36]	IL-10 (pg/mL)	↑	1 h
14	Karottki et al. [23]	Leukocytes (×10^9^ cells/L) (indoor exposure)	↑	at the end of the 2-day indoor air monitoring period (indoor exposure)
15	Karottki et al. [20]	Leukocytes (10^9^ cells/L)	↓	hours–days (outdoor exposure)
16	Karottki et al. [20]	Lymphocytes (×10^9^ cells/L) (indoor exposure)	↑	at the end of the 2-day indoor air monitoring period (indoor exposure)
17	Karottki et al. [20]	Monocytes (×10^9^ cells/L) (indoor exposure)	↑	at the end of the 2-day indoor air monitoring period (indoor exposure)
18	Jiang et al. [41]	TNF-α (pg/mL)	↑	0–3 h
19	Kumarathasan et al. [28]	Fibrinogen	↑	data not provided
20	Jiang et al. [41]	HDL (mmol/L)	↓	13–24 h
21	Jiang et al. [41]	LDL (mmol/L)	↑	7–12 h
22	Kumarathasan et al. [28]	A2M	↑	blood samples (*n* = 52) were collected late in the afternoon (between 2 and 5 pm) at the end of the exposure week (Friday) at both College and Bayview sites; the baseline sample was collected for blood one week prior (Friday between 2 pm and 5 pm) to the beginning of the sequence of exposures
23	Kumarathasan et al. [28]	Adipsin	↑	
24	Kumarathasan et al. [28]	AGP	↑	
25	Kumarathasan et al. [28]	Haptoglobin	↑	
26	Kumarathasan et al. [28]	L-selectin	↑	
27	Kumarathasan et al. [28]	PF4	↑	
28	Kumarathasan et al. [28]	ET 1–21 (plasma)	↑	
29	Karottki et al. [20]	MVF (outdoor exposure)	↓	at the end of the 2-day indoor air monitoring period (indoor exposure)
30	Karottki et al. [20]	HbA1c (mmol/mol)	↑	at the end of the 2-day indoor air monitoring period (indoor exposure)
31	Jiang et al. [41]	Glucose (mmol/L)	↑	0–3 h
32	Jiang et al. [41]	Insulin (pmol/L)	↑	0–3 h

* Long-term studies.

## Supplementary Materials

The following supporting information can be downloaded at https://www.mdpi.com/article/10.3390/jcm14082846/s1.
