# Supplementary material for: Short-Term and Long-Term Effects of Inhaled Ultrafine Particles on Blood Markers of Cardiovascular Diseases: A Systematic Review and Meta-Analysis
Source: J Clin Med. 2025 Apr 21;14(8):2846. doi: 10.3390/jcm14082846 (PMC12028172; doi:10.3390/jcm14082846)
Supplement: Supplementary file 1 [file jcm-14-02846-s001.zip › jcm-3504579-supplementary.pdf]

Short-term and long-term effects of inhaled ultrafine particles on blood markers of cardiovascular diseases: A systematic review and meta-analysis

Joanna I. Lachowicz,[1,*] Pawel Gac[1]

[1]*Faculty of Health Sciences, Department of Environmental Health, Occupational Medicine and Epidemiology, Division of Environmental Health, Occupational Medicine and Epidemiology, Wroclaw Medical University, Mikulicza-Radeckiego 7, Wroclaw, PL 50-368, Poland.*

Text S1. Search terms used in literature search..2

Text S2. Standardization of the effect estimates..3

Table S1. Eligibility criteria of long-term exposures on UPFs. 4

Table S2. Eligibility criteria of short-term exposures on UPFs. 6

Table S3. Risk of Bias (RoB) assessment prepared according to "Risk of Bias assessment instrument for systematic reviews informing WHO Global Air Quality Guidelines". By: the WHO Global Air Quality Guidelines Working Group on Risk of Bias Assessment..7

Table S4. Main characteristics of blood markers unbalance upon UFPs exposure. (*) Studies analyzing long-term effects of UFPs exposure on blood markers of cardiovascular diseases. 9

Text S1. Search terms used in literature search.

Search terms used on *PubMed* were: “Nanoparticles" OR "Ultrafine particles" AND “Cardiovascular” AND “Exposure” AND “blood markers”.

Search terms used on *Web of Science* were: “Nanoparticles" OR "Ultrafine particles" AND “Cardiovascular” AND “Exposure” AND “blood markers”.

Search terms used on *Embase* were: “Nanoparticles" OR "Ultrafine particles" AND “Cardiovascular” AND “Exposure” AND “blood markers”.

Search terms used on *Scopus* were: “Nanoparticles" OR "Ultrafine particles" AND “Cardiovascular” AND “Exposure” AND “blood markers”.

Text S2. Standardization of the effect estimates.

When BP indices were not transformed:

$$\text{Percent change} = \beta \,/\, \text{mean} \times 100\%$$

$$95\%\ \text{CI} = (\beta \pm 1.96 \times \text{SE}) \,/\, \text{mean} \times 100\%$$

β is the estimated regression coefficient increase in UFPs (expressed in PNC), *SE* is the standard error of *β*, *mean* is the arithmetic mean of the examined BP index (SBP or DBP).

We chose transformation to percent changes in the geometric mean because we were not able to calculate percent changes in the arithmetic mean in all studies due to missing geometric or/and arithmetic mean of the BP indices. The procedure above enabled us to include all eligible articles in meta-analyses.

Table S1. Eligibility criteria of long-term exposures on UPFs.

PECOS	Inclusion	Exclusion
**P**opulation	General human population (including subgroups: children, women, man) of all ages, living in developed and developing areas, both urban and rural. No geographical restrictions	No exclusion criteria applied
	Exposure to UFPs via inhalation through ambient air (this covers exposures in both outdoor and indoor environments)	

**E**xposure	Long-term exposure (in the order of months to years) to ambient air UFPs a concentration unit (particles/m^3)	No exclusion criteria applied
	Additionally, Long-term exposure to other air pollutants	
**C**omparator	Exposure to lowest levels of the air UFPs in the same or a control population	No exclusion criteria applied
**O**utcome	Health outcomes selected in relation to long-term exposure include: blood markers of CVDs	No exclusion criteria applied
**S**tudy design	Human epidemiological studies such as:<br>- panel studies<br>- cohort studies<br>- cross-sectional studies<br>- cross-over studies<br>- Published (or accepted for publication, i.e. in press) studies in peer-reviewed indexed journals in any language (abstract in English language)	Qualitative studies; Studies without individual- level data, that is, fully group-level (ecological) covariates; Reviews and methodological papers; Non-human studies (in vivo, in vitro, other);

Table S2. Eligibility criteria of short-term exposures on UPFs.

PECOS	Inclusion	Exclusion
**P**opulation	General human population (including subgroups: children, women, man) of all ages, living in developed and developing areas, both urban and rural. No geographical restrictions	No exclusion criteria applied
	Exposure to UFPs via inhalation through ambient air (this covers exposures in both outdoor and indoor environments)	
**E**xposure	Short-term exposure (in the order of hours to 7 days) to ambient air UFPs a concentration unit (particles/m^3)	No exclusion criteria applied
	Additionally, short-term exposure to other air pollutants	
**C**omparator	Exposure to lowest levels of the air UFPs in the same or a control population	No exclusion criteria applied
**O**utcome	Health outcomes selected in relation to short-term exposure include: blood markers of CVDs	No exclusion criteria applied
**S**tudy design	Human epidemiological studies such as:<br>- panel studies<br>- cohort studies<br>- cross-sectional studies<br>- cross-over studies<br>- Published (or accepted for publication, i.e. in press) studies in peer-reviewed indexed journals in any language (abstract in English language)	Qualitative studies; Studies without individual-level data, that is, fully group-level (ecological) covariates; Reviews and methodological papers; Non-human studies (in vivo, in vitro, other);

Table S3. Risk of Bias (RoB) assessment prepared according to "Risk of Bias assessment instrument for systematic reviews informing WHO Global Air Quality Guidelines". By: the WHO Global Air Quality Guidelines Working Group on Risk of Bias Assessment.

		Confounding				Selection bias		Exposure assessment					Outcome measurement				Missing data		
N^0	Name	Were all confounders considered adjusted for in the analysis?	Validity of measuring of confounding factors	Control in analysis (Did the authors use an appropriate analysis method or study design that controlled for confounding domains?)	**Overall**	Selection of participants into the study (includes non-response)	**Overall**	Methods used for exposure assessment	Exposure measurement methods comparable across the range of exposure	Change in exposure status (for long-term studies only)	Exposure contrast	**Overall**	Blinding of outcome measurement	Validity of outcome measurements	Outcome measurement	**Overall**	Missing data of outcome measures	Missing data of exposures	**Overall**
1	Brugge et al [40]	high	high	high	high	low	low	low	low	moderate	moderate	moderate	low	low	low	low	low	low	low
2	Meier et al.[21]	low	low	low	low	moderate	moderate	low	low	NA	low	low	low	low	low	low	low	low	low
3	Karottki 2014 et al.[29]	moderate	moderate	moderate	moderate	moderate	moderate	moderate	moderate	NA	low	moderate	low	low	low	low	low	low	low
4	Devlin et al. [38]	high	high	high	high	moderate	moderate	low	low	NA	low	low	low	low	low	low	low	low	low
5	Padró-Martínez et al. [43]	high	high	high	high	moderate	moderate	low	low	NA	low	low	low	low	low	low	low	low	low
6	Karottki 2015 et al. [31]	moderate	moderate	moderate	moderate	moderate	moderate	low	low	NA	low	low	low	low	low	low	low	low	low
7	Fuller et al. [30]	high	high	high	high	low	low	low	low	NA	low	low	low	low	low	low	low	low	low
8	Shvedova et al. [24]	moderate	moderate	moderate	moderate	moderate	moderate	moderate	moderate	NA	moderate	moderate	low	low	low	low	low	low	low

9	Liu et al. [26]	low	low	low	low	low	low	low	low	NA	low	low	low	low	low	low	low	low	low
10	Corlin et al. [44]	high	high	high	high	low	low	moderate	low	NA	low	low	low	low	low	low	low	low	low
11	Kumarathasan et al. [32]	low	low	low	low	moderate	moderate	low	low	NA	low	low	low	low	low	low	low	low	low
12	Pilz et al. [41]	low	low	low	low	low	low	low	low	moderate	low	low	low	low	low	low	low	low	low
13	Espín-Perez et al. [33]	low	low	low	low	low	low	low	low	NA	low	low	low	low	low	low	low	low	low
14	Krauskopfa et al. 34]	low	low	low	low	moderate	moderate	low	low	NA	low	low	low	low	low	low	low	low	low
15	Mancini et al. [27]	moderate	moderate	moderate	moderate	moderate	moderate	low	low	NA	low	low	low	low	low	low	low	low	low
16	Guo et al. [23]	high	high	high	high	high	high	ND	ND	NA	ND	ND	low	low	low	low	low	high	high
17	Bello et al. [25]	moderate	moderate	moderate	moderate	moderate	moderate	low	low	NA	low	low	low	low	low	low	low	low	low
18	Du et al. 2022 [39]	low	low	low	low	low	low	low	low	NA	low	low	low	low	low	low	low	low	low
19	Zhang et al. [36][36]	low	low	low	low	low	low	low	low	NA	low	low	low	low	low	low	low	low	low
20	Yao et al. [45]	low	low	low	low	low	low	low	low	low	low	low	low	low	low	low	low	low	low
21	Roswell et al. 37]	low	low	low	low	low	low	moderate	low	low	low	low	low	low	low	low	low	low	low
22	Du et al. 2023 [35]	low	low	low	low	moderate	moderate	low	low	NA	low	low	low	low	low	low	low	low	low
23	Vogli et al. [42]	low	low	low	low	low	low	moderate	moderate	moderate	moderate	moderate	low	low	low	low	low	low	low
24	Jiang et al. [28]	low	low	low	low	moderate	moderate	low	low	NA	low	low	low	low	low	low	low	low	low

Table S4. Main characteristics of blood markers unbalance upon UFPs exposure. (*) Studies analyzing long-term effects of UFPs exposure on blood markers of cardiovascular diseases.

No	Study	Name of marker	Mean (SD) / Median Concentration (5th, 95th percentiles)*	N	Estimate value	Lower	Upper	SE	Estimate type	Measure
									**INFLAMMATION**	
1	Karottki et al. [29]	CD11b %	67.8 (32.9, 84)*	78	-4.4	-8.3	-0.3	2.04	Percent changes (95% confidence interval) in outcome levels associated with one interquartile range (IQR) increase in indoor and outdoor exposures	at the end of the 2-day indoor air monitoring period (indoor exposure)
2	Karottki et al. [29]	CD31 %	92.9 (82.1, 97.9)*	48	0.0	-1.1	1.1	0.6		Hours-days (indoor exposure)
3	Karottki et al. [29]	CD31 %	92.9 (82.1, 97.9)*	48	-0.2	-1.9	1.5	0.9		Hours-days (outdoor exposure)
4	Karottki et al. [29]	CD62 %	62.4 (41.9, 79)*	48	0.2	-2.4	3.0	1.4		Hours-days (indoor exposure)
5	Karottki et al. [29]	CD62 %	62.4 (41.9, 79)*	48	-0.7	-3.7	2.4	1.6		Hours-days (outdoor exposure)
6	Karottki et al. [29]	CD11b %	38.3 (6.7, 70.0)*	48	1.6	-6.2	10.1	4.2		Hours-days (indoor exposure)
7	Karottki et al. [29]	CD11b %	38.3 (6.7, 70.0)*	48	4.3	-3.7	13.0	4.3		Hours-days (outdoor exposure)
8	Karottki et al. [29]	CD49 %	71.7 (32.6, 95.6)*	48	-0.8	-4.2	2.7	1.8		Hours-days (indoor exposure)
9	Karottki et al. [29]	CD49 %	71.7 (32.6, 95.6)*	48	1.0	-3.4	5.7	2.3		Hours-days (outdoor exposure)
10	Karottki et al. [29]	CC16 (ng/mL)	4.0 (2.0, 9.4)*	48	-0.7	-5.1	3.9	2.3		Hours-days (indoor exposure)
11	Karottki et al. [29]	CC16 (ng/mL)	4.0 (2.0, 9.4)*	48	2.1	-2.8	7.3	2.6		Hours-days (outdoor exposure)
12	Zhang et al.	Granulocyte-macrophage colony-stimulating factor (pg/mL)	0.15 (-0.21-0.73)*	56	0.5	0.0	0.9	0.2	Changes or percentage changes of inflammatory biomarkers associated with an IQR increase of traffic-related air pollutants	1 hour
13	Karottki et al. [29]	Granulocytes (10^9 cells/L)	2.9 (1.8, 5.4)*	48	0.2	-3.4	3.9	1.9	Percent changes (95% confidence interval) in outcome levels associated with one interquartile range increase (IQR)	Hours-days (indoor exposure)
14	Karottki et al. [29]	Granulocytes (10^9 cells/L)	2.9 (1.8, 5.4)*	48	0.4	-3.4	4.4	2.0		Hours-days (outdoor exposure)
15	Zhang et al. [36]	Interferon-induced T-cell alpha chemoattractant (pg/mL)	0.97 (-1.24-3.16)*	56	2.5	0.5	4.6	1.1	Changes or percentage changes of inflammatory biomarkers associated with an IQR increase of traffic-related air pollutants	1 hour
16	Zhang et al. [36]	IFN-γ (pg/mL)	1.39 (-7.51-6.33)*	56	1.9	-4.8	8.5	3.4		
17	Jiang et al.[28]	IFN-γ (pg/mL)	11.6 (3.6); 11.2 (9.0-13.4)*	32	8.5	0.8	16.7	4.0	Estimated percent changes or changes (mean and 95% CIs) in circulating biomarkers associated with each interquartile range increase in UFP concentrations in two-pollutant model	0-3 hours
18	Bello et al. [25]	IL-1α		145	3.1			1.5	β-estimates (β × 10-5)	two randomly selected consecutive weeks: Monday AM and Friday PM on both Week 1 and Week 2 during 2018–2021
19	Zhang et al. [36]	IL-1β (pg/mL)	0.04 (-0.02,0.15)*	56	0.1	0.0	0.2	0.1	Changes or percentage changes of inflammatory biomarkers associated with an IQR increase of traffic-related air pollutants	1 hour

20	Bello et al. [25]	IL-1β		145	2.9			1.3	β-estimates (β × 10-5)	two randomly selected consecutive weeks: Monday AM and Friday PM on both Week 1 and Week 2 during 2018–2019
21	Jiang et al. [28]	IL-1β (pg/mL)	6.3 (1.8); 6.2 (5.4-6.9)*	32	19.9	3.8	38.5	8.8	Estimated percent changes or changes (mean and 95% CIs) in circulating biomarkers associated with each interquartile range increase in UFP concentrations in two-pollutant model	0-3 hours
22*	Padró-Martínez et al. [43]	IL-6		20	-19.0	-35.0	-1.0	8.7	Mean change (%) and 95% confidence intervals (CI)	10^4 particles/cm3 increase in PNC moving average (MA) (14- or 21-day MA) adjusted for baseline blood biomarker concentrations.
23	Fuller et al. [30]	IL-6 (pg/mL)	3.68 (7.91); 1.6 (0.95-3.24)*	142	-1.7	-14.5	13.1	7.0	Estimated percent change (95% CI)	two time points approximately 5 months apart
24	Zhang et al. [36]	IL-6 (pg/mL)	0.22 (-0.06-0.43)*	56	-0.2	-0.7	0.2	0.2	Changes or percentage changes of inflammatory biomarkers associated with an IQR increase of traffic-related air pollutants	1 hour
25	Jiang et al. [28]	IL-6 (pg/mL)	2.9 (1.5); 2.7 (2.1-3.4)*	32	16.3	5.5	28.3	5.8	Estimated percent changes or changes (mean and 95% CIs) in circulating biomarkers associated with each interquartile range increase in UFP concentrations in two-pollutant model	0-3 hours
26*	Brugge et al. [40]	IL-6 (pg/mL)	2.91 (2.77); 1.63*	260	0.5	0.0	1.2	0.3	%Diff. Regression models. Adj. R2 = 0.05; 95%CI. Distance from Highway <50m	in the morning in the study areas
27*	Brugge et al. [40]	IL-6 (pg/mL)	3.27 (6.04); 1.79*	260	0.5	0.1	1.1	2.5	%Diff. Regression models. Adj. R2 = 0.05; 95%CI. Distance from Highway 150-250 m	
28	Meier et al. [21]	IL-7 (ng/L)	0.54 (0.29)	18					Linear mixed-effects regression models	5-day UFP exposure, no correlation
29	Jiang et al. [28]	IL-8 (pg/mL)	15.2 (11.0); 11.7 (8.7-17.5)*	32	12.4	0.9	25.1	6.2	Estimated percent changes or changes (mean and 95% CIs) in circulating biomarkers associated with each interquartile range increase in UFP concentrations in two-pollutant model	0-3 hours
30	Zhang et al. [36]	IL-8 (pg/mL)	-0.35 (-1.76-1.63)*	56	0.4	-0.6	1.3	0.5	Changes or percentage changes of inflammatory biomarkers associated with an IQR increase of traffic-related air pollutants	1 hour
31	Zhang et al. [36]	IL-10 (pg/mL)	0 (-0.40-0.26)*	56	0.3	0.0	0.5	0.1		
32	Zhang et al. [36]	IL-17A (pg/mL)	-0.05 (-0.38-0.14)*	56	0.0	-0.4	0.3	0.2		
33	Zhang et al. [36]	IL-23 (pg/mL)	0 (-10.31-10.34)*	56	-0.1	-13.4	7.2	5.2		
34	Karottki et al. [29]	Leukocytes ($\times 10^9$ cells/L) (indoor exposure)	5.4 (3.8, 7.7)*	78	3.5	0.2	6.9	1.7	Percent changes (95% confidence interval) in outcome levels associated with one interquartile range (IQR) increase in indoor and outdoor exposures	at the end of the 2-day indoor air monitoring period; (indoor exposure)
35	Karottki et al. [29]	Leukocytes (10^9 cells/L)	5.7 (3.9, 8.3)*	48	-0.6	-3.3	2.2	1.4		Hours-days, (indoor exposure)
36	Karottki et al. [29]	Leukocytes (10^9 cells/L)	5.7 (3.9, 8.3)*	48	-0.8	-1.8	-2.1	-0.1		Hours-days (outdoor exposure)
37	Karottki et al. [29]	Lymphocytes ($\times 10^9$ cells/L) (indoor exposure)	2.0 (1.2, 3.1)*	78	6.6	2.0	11.5	2.4		at the end of the 2-day indoor air monitoring period (indoor exposure)
38	Karottki et al. [29]	Lymphocytes (10^9 cells/L)	2.0 (0.9, 3.3)*	48	-1.7	-5.3	2.1	1.9		Hours-days (indoor exposure)
39	Karottki et al. [29]	Lymphocytes (10^9 cells/L)	2.0 (0.9, 3.3)*	48	1.7	-1.6	5.0	1.7		Hours-days (outdoor exposure)
40	Karottki et al. [29]	Monocytes (×109 cells/L) (indoor exposure)	0.6 (0.4, 0.8)*	78	4.1	0.4	7.9	1.9		at the end of the 2-day indoor air monitoring period (indoor exposure)
41	Karottki et al. [29]	Monocytes (10^9 cells/L)	0.6 (0.4, 0.9)*	48	-1.4	-4.3	1.5	1.5		Hours-days (indoor exposure)
42	Karottki et al. [29]	Monocytes (10^9 cells/L)	0.6 (0.4, 0.9)*	48	2.0	-0.6	4.8	1.4	Percent changes (95% confidence interval) in outcome levels associated with one interquartile range increase (IQR)	Hours-days (outdoor exposure)
43	Fuller et al. [30]	TNF-RII (pg/mL)	3013 (1499); 2630 (2144-3317)*	142	1.9	-3.5	7.7	2.8	Estimated percent change (95% CI)	two time points approximately 5 months apart
44	Meier et al. [21]	TNFα (ng/L)	3.79 (1.00)	18					Linear mixed-effects regression models	5-day UFP exposure, no correlation
45	Zhang et al. [36]	TNF-α (pg/mL)	-0.01 (-0.41-0.42)*	56	0.0	-0.3	0.2	0.1	Changes or percentage changes of inflammatory biomarkers associated with an IQR increase of traffic-related air pollutants	1 hour
46	Jiang et al. [28]	TNF-α (pg/mL)	9.3 (6.4); 7.3 (5.0-10.9)*	32	34.7	15.6	56.8	10.5	Estimated percent changes or changes (mean and 95% CIs) in circulating biomarkers associated with each interquartile range increase in UFP concentrations in two-pollutant model	0-3 hours
**COAGULATION**										

47	Kumarathasan et al. [32]	Fibrinogen		52	0.5	0.3	0.8	0.1	Relative change in target biomarker levels associated with IQR changes in UFPs	data no provided
48*	Vogli et al. [42]	Fibrinogen (g/L)	2.6 (0.6)	4261	0.7				Effect estimates and 95 % CI of the associations between long-term exposure to air pollution and biomarkers of inflammation and coagulation, per IQR in air pollutant for the main confounder model	On the day of the examination, participants' blood samples were collected
49	Fuller et al. [30]	Fibrinogen (mg/dL)	395.7 (86.5); 383.5 (332.5-436.0)*	142	-0.5	-16.3	15.2	8.0	Estimated percent change (95% CI)	two time points approximately 5 months apart
50	Devlin et al. [38]	Plasminogen		34	-13.8			5.8	% Changes in Soluble Blood Markers (20 h/pre) Associated with Particle Number ± standard error following UCAPS exposure compared with exposure to clean air	participants were exposed twice, while at rest for a 2-h period: once to clean air and once to concentrated ambient ultrafine particles.
51	Devlin et al. [38]	Thrombomodulin		34	-1.8			0.9		
**LIPID**										
52	Roswell et al. [37]	HDL		32,851	0.0	0.0	0.0		β-estimates	24-h UFP-exposure; per 10,000 particles/cm3
53	Roswell et al. [37]	HDL		32,851	0.0	0.0	0.0			72-h UFP-exposure; per 10,000 particles/cm3
54	Roswell et al. [37]	HDL		32,851	0.0	0.0	0.0			7-day UFP-exposure; per 10,000 particles/cm3
55	Roswell et al. [37]	HDL		32,851	0.0	0.0	0.0			30-day UFP-exposure; per 10,000 particles/cm3
56	Roswell et al. [37]	HDL		32,851	0.0	0.0	0.0			90-day UFP-exposure; per 10,000 particles/cm3
57	Jiang et al. [28]	HDL (mmol/L)	1.5 (0.3); 1.4 (1.2-1.7)*	32	-3.7	-6.6	-0.8	1.5	Estimated percent changes or changes (mean and 95% CIs) in circulating biomarkers associated with each interquartile range increase in UFP concentrations in two-pollutant model	13-24 hours
58	Roswell et al. [37]	non-HDL		32,851	0.0	0.0	0.0		β-estimates	24-h UFP-exposure; per 10,000 particles/cm3
59	Roswell et al. [37]	non-HDL		32,851	0.0	0.0	0.0			72-h UFP-exposure; per 10,000 particles/cm3
60	Roswell et al. [37]	non-HDL		32,851	0.0	0.0	0.1			7-day UFP-exposure; per 10,000 particles/cm3
61	Roswell et al. [37]	non-HDL		32,851	0.1	0.0	0.1			30-day UFP-exposure; per 10,000 particles/cm4
62	Roswell et al. [37]	non-HDL		32,851	0.0	-0.1	0.1			90-day UFP-exposure; per 10,000 particles/cm3
63	Jiang et al. [28]	LDL (mmol/L)	2.2 (0.5); 2.2 (1.8-2.6)*	32	6.4	0.5	12.6	3.1	Estimated percent changes or changes (mean and 95% CIs) in circulating biomarkers associated with each interquartile range increase in UFP concentrations in two-pollutant model	7-12 hours
**OTHER**										
64	Meier et al. [21]	Plasma vWF (%)	109.22 (39.57)	18					Linear mixed-effects regression models	5-day UFP exposure, no correlation
65	Meier et al. [21]	Plasma tissue factor (ng/L)	74.84 (29.77)	18						5-day UFP exposure, no correlation
66	Kumarathasan et al. [32]	A2M		52	0.5	0.3	0.8	0.1	Relative change in target biomarker levels associated with IQR changes in UFPs	blood samples (n = 52) were collected late in the afternoon (between 2 and 5 pm) at the end of the exposure week (Friday) at both College and Bayview sites. Baseline sample was collected for blood one week prior (Friday between 2 pm and 5 pm) to the beginning of the sequence of exposures.
67	Kumarathasan et al. [32]	Adipsin		52	0.2	0.1	0.6	0.1		
68	Kumarathasan et al. [32]	AGP		52	0.4	0.2	0.6	0.1		
69	Kumarathasan et al. [32]	Haptoglobin		52	0.5	0.4	0.8	0.1		
70	Kumarathasan et al. [32]	L Selectin		52	0.7	0.5	0.9	0.1		
71	Kumarathasan et al. [32]	PF4		52	0.5	0.3	0.8	0.1		
72	Kumarathasan et al. [32]	ET 1–21 (plasma)		52	4.4	1.1	17.0	4.1		
73	Karottki et al. [31]	MVF (outdoor exposure)	1.77 (1.14, 2.87)*	78	-8.4	-15.2	-1.0	3.6	Percent changes (95% confidence interval) in outcome levels associated with one interquartile range (IQR) increase in indoor and outdoor exposures	at the end of the 2-day indoor air monitoring period, (indoor exposure)
74	Karottki et al. [31]	HbA1c (mmol/mol)	36 (30, 42)*	78	2.1	0.9	3.3	0.6	Percent changes (95% confidence interval) in outcome levels associated with one interquartile range (IQR) increase in indoor and outdoor exposures	at the end of the 2-day indoor air monitoring period, (indoor exposure)

75	Bello et al. [25]	Fractalkine	1.24	19	3.1			1.24	β-estimates (β × 10-5)	two randomly selected consecutive weeks: Monday AM and Friday PM on both Week 1 and Week 2 during 2018–2020
76	Devlin et al. [38]	SAA (mg/L)		34	77.5			37.2	% Changes in Soluble Blood Markers (20 h/pre) Associated with Particle Number ± standard error following UCAPS exposure compared with exposure	participants were exposed twice, while at rest for a 2-h period: once to clean air and once to concentrated ambient ultrafine particles.
77	Meier et al. [21]	SAA (mg/L)	4.63 (4.01)	18					Linear mixed-effects regression models	5-day UFP exposure, no correlation
78	Jiang et al. [28]	Glucose (mmol/L)	4.8(0.5)	32	3.9	0.9	6.9	1.5	Estimated percent changes or changes (mean and 95% CIs) in circulating biomarkers associated with each interquartile range increase in UFP concentrations in two-pollutant model	0-3 hours
79	Jiang et al. [28]	Insulin (pmol/L)	108.2 (95.6)	32	24.3	3.5	49.2	11.7	Estimated percent changes or changes (mean and 95% CIs) in circulating biomarkers associated with each interquartile range increase in UFP concentrations in two-pollutant model	0-3 hours
80	Jiang et al. [28]	ApoA-I (g/L)	1.5(0.2)	32	-1.7	-3.4	0.2	0.9	Estimated percent changes or changes (mean and 95% CIs) in circulating biomarkers associated with each interquartile range increase in UFP concentrations in two-pollutant model	25-48 hours
81	Jiang et al. [28]	ApoB	0.7(0.1)	32	4.7	-0.1	9.7	2.5	Estimated percent changes or changes (mean and 95% CIs) in circulating biomarkers associated with each interquartile range increase in UFP concentrations in two-pollutant model	25-48 hours
